# Tracking the Antigenic Evolution of Foot-and-Mouth Disease Virus

**DOI:** 10.1371/journal.pone.0159360

**Published:** 2016-07-22

**Authors:** Richard Reeve, Daryl W. Borley, Francois F. Maree, Sasmita Upadhyaya, Azwidowi Lukhwareni, Jan J. Esterhuysen, William T. Harvey, Belinda Blignaut, Elizabeth E. Fry, Satya Parida, David J. Paton, Mana Mahapatra

**Affiliations:** 1 Boyd Orr Centre for Population and Ecosystem Health, Institute of Biodiversity, Animal Health and Comparative Medicine, College of Medical, Veterinary and Life Sciences, University of Glasgow, Glasgow, United Kingdom; 2 The Pirbright Institute, Pirbright, Woking, Surrey, United Kingdom; 3 Division of Structural Biology, University of Oxford, The Henry Wellcome Building for Genomic Medicine, Headington, Oxford, United Kingdom; 4 ARC-Onderstepoort Veterinary Institute, Transboundary Animal Diseases Programme, Onderstepoort, South Africa; 5 Department of Microbiology and Plant Pathology, Faculty of Agricultural and Natural Sciences, University of Pretoria, Pretoria, South Africa; 6 Department of Production Animal Studies, Faculty of Veterinary Science, University of Pretoria, Onderstepoort, South Africa; Centro de Biología Molecular Severo Ochoa (CSIC-UAM), SPAIN

## Abstract

Quantifying and predicting the antigenic characteristics of a virus is something of a holy grail for infectious disease research because of its central importance to the emergence of new strains, the severity of outbreaks, and vaccine selection. However, these characteristics are defined by a complex interplay of viral and host factors so that phylogenetic measures of viral similarity are often poorly correlated to antigenic relationships. Here, we generate antigenic phylogenies that track the phenotypic evolution of two serotypes of foot-and-mouth disease virus by combining host serology and viral sequence data to identify sites that are critical to their antigenic evolution. For serotype SAT1, we validate our antigenic phylogeny against monoclonal antibody escape mutants, which match all of the predicted antigenic sites. For serotype O, we validate it against known sites where available, and otherwise directly evaluate the impact on antigenic phenotype of substitutions in predicted sites using reverse genetics and serology. We also highlight a critical and poorly understood problem for vaccine selection by revealing qualitative differences between assays that are often used interchangeably to determine antigenic match between field viruses and vaccine strains. Our approach provides a tool to identify naturally occurring antigenic substitutions, allowing us to track the genetic diversification and associated antigenic evolution of the virus. Despite the hugely important role vaccines have played in enhancing human and animal health, vaccinology remains a conspicuously empirical science. This study advances the field by providing guidance for tuning vaccine strains via site-directed mutagenesis through this high-resolution tracking of antigenic evolution of the virus between rare major shifts in phenotype.

## 1. Introduction

Foot-and-mouth disease (FMD) is a highly contagious disease, predominantly affecting animals of the order artiodactyla, with the primary domestic hosts being cattle, buffalo (*Bubalus bubalus*), sheep, pigs and goats, although the causative virus also circulates in wildlife, in particular in the African buffalo (*Syncerus caffer*) [1, 2]. Globally, it is one of the most economically important livestock diseases and is still widely distributed. Although mortality is generally low in adult animals, high mortality rates have been reported in very young animals and in some wildlife species [3–5]. However, production losses in FMD-affected domestic animals are high and the multiplicity of serotypes and strains allows repeated infection to occur [6]. The developed world has therefore mainly eradicated FMD and restricts trade from regions where it still occurs. This damages the economies of enzootically infected countries [7] whilst incursions into previously FMD-free countries are very costly; for example, in the 2001 outbreak of FMD in the UK approximately 4 million animals were slaughtered to control the disease with compensation payments of £1.4 billion to farmers and other substantial indirect losses [8]. Recent outbreaks in the Far East, North Africa and Eastern Europe [9] demonstrate the continuing dissemination of FMD into areas previously free from disease, with major economic impact.

The disease is caused by foot-and-mouth disease virus (FMDV), a single-stranded positive-sense RNA virus belonging to the genus *Aphthovirus* in the family *Picornaviridae*. Due to the lack of proof reading ability of the FMDV RNA polymerase, novel genetic and antigenic variants continue to emerge within each of the six recently observed serotypes of the virus, limiting the strength of cross-protective immunity between some strains, even within the same serotype. This has so far confounded attempts to develop vaccines that provide a broad range of protection against viruses both within and between serotypes. Such broad-range protection is one of the major goals underpinning current research in FMDV vaccine development, making it vitally important to identify those areas of the capsid that are targets for protective immunity.

Antibodies play a critical role in the host response to FMDV infection [10, 11] and generally, levels of neutralising antibody correlate well with protection observed *in vivo* [12, 13]. However, the interaction between FMDV and the humoral immune system is complex. Phylogenetically distant viruses can be antigenically close, with some field viruses exhibiting higher antibody reactivity than the homologous virus to which the antiserum had been raised [14]. Other studies have shown that individual mutations may have a large effect on virus antigenicity [15, 16]. Investigation of FMDV serotype C showed that fluctuations among a small number of residues drove antigenic diversity [17]. This work suggested that the antigenic drift of FMDV does not occur through the gradual accumulation of amino acid changes across the entire surface of the virus, but through changes in a small number of immunodominant residues within antigenic sites. However, these conclusions were derived from studies of only one of the three surface-exposed structural proteins. Previous work looking at the whole capsids of naturally occurring isolates could only identify multiple co-occurring substitutions [18].

The study of monoclonal antibody (mAb) escape mutants has instead helped to identify a set of residues that drive antigenic diversity in FMDV, with five antigenic sites, each containing multiple residues, previously reported for serotype O [15, 19] and three for serotype SAT1 [20]. There is experimental evidence that identification and modification of such sites can help increase cross-reactivity of vaccines. For example, it has been shown in serotype C that removing two immunodominant antigenic sites increases the cross-reactivity of antiserum harvested from infected mice [21]. However, there is evidence that antigenic sites that are targeted by antibodies in convalescent sera remain to be identified. Examination of a five-site neutralisation escape mutant of serotype O virus showed cross-protection and residual *in vitro* neutralisation by antisera generated against the parental virus [22], suggesting that other capsid residues apart from the known neutralising epitopes play a role in the antigenicity of serotype O viruses, and tools to identify such sites would be very valuable.

Reverse genetics allows the definitive identification of the antigenic impact in specific viruses of substitutions previously observed during the evolution of the virus. This approach has recently identified that the largest effects in the transitions between antigenic clusters in Influenza A (H3N2) can be reproduced by single amino acid substitutions at just 7 positions near the receptor-binding site of the haemaggluttinin protein [23]. This was possible for human influenza because the search was limited to substitutions fixed in the trunk lineage, from which all future viruses descend [24], unlike the wide diversification that FMDV has undergone [17].

We have recently developed methods to identify regions containing epitopes directly from serology by correlating amino acid changes on the capsid with serological reactivity changes between multiple FMDV virus pairs [25, 26]. This allows examination of the interactions between viruses and the polyclonal antibody responses of a natural host and therefore provides advantages over traditional methods for epitope mapping using mouse mAbs and virus escape mutants; the residues identified are naturally occurring changes reflecting the evolution of FMDV in the field. We cannot exclude the possibility that some of the identified residues may have been introduced during growth in cell culture, but this is a feature that affects epitope mapping using mouse mAbs as well. The technique also offers advantages over using reverse genetics to introduce point mutations, in that it provides simultaneous identification of multiple antigenic sites, each with at least two points of independent evidence across the phylogeny. Nonetheless, sites identified by our method can be validated by the introduction of such point mutations.

Here, we extend our approach to study an extensive set of FMD viruses of serotypes O and SAT1, which cause more than 70% of FMD outbreaks worldwide [27, 28]. We identify residues that form part of epitopes for both serotypes, and we compare these to residues identified by studies of mAb resistant (mar-) mutants to validate the majority of the sites; where such mutants do not exist, we used reverse genetics to engineer viruses with appropriate mutations instead. Using the identified substitutions, we build antigenic phylogenies of these serotypes, reconstructing key parts of the antigenic history of FMDV.

Nevertheless, such results remain dependent on the relationship between the assay and protection in the field–for FMDV the dominant assays used for this purpose are Virus Neutralisation Tests (VNTs) and Liquid Phase Blocking ELISAs (LPBEs), which are well correlated with respect to homologous protection [29, 30]. We directly compare the perceived antigenic evolution of the virus as revealed by VNTs and LPBEs to investigate differences between the assays in defining heterologous (cross-)protection.

## 2. Results

### 2.1 Virus neutralization and ELISA titres

We selected 103 serotype O viruses and 26 serotype SAT1 viruses (S1 Table). Cattle antisera were obtained against seven O and four SAT1 strains. VNTs and LPBEs were each carried out using five of the O antisera (for a total of 324 and 317 pairs of virus and antiserum, respectively), with an overlap of three. VNTs were carried out with all four SAT1 antisera (for a total of 100 pairs). The latter results were combined with previous data [25] to provide 43 viruses and 153 pairs for analysis.

### 2.2 Sequencing and phylogenetic analysis

**Fig 1:**
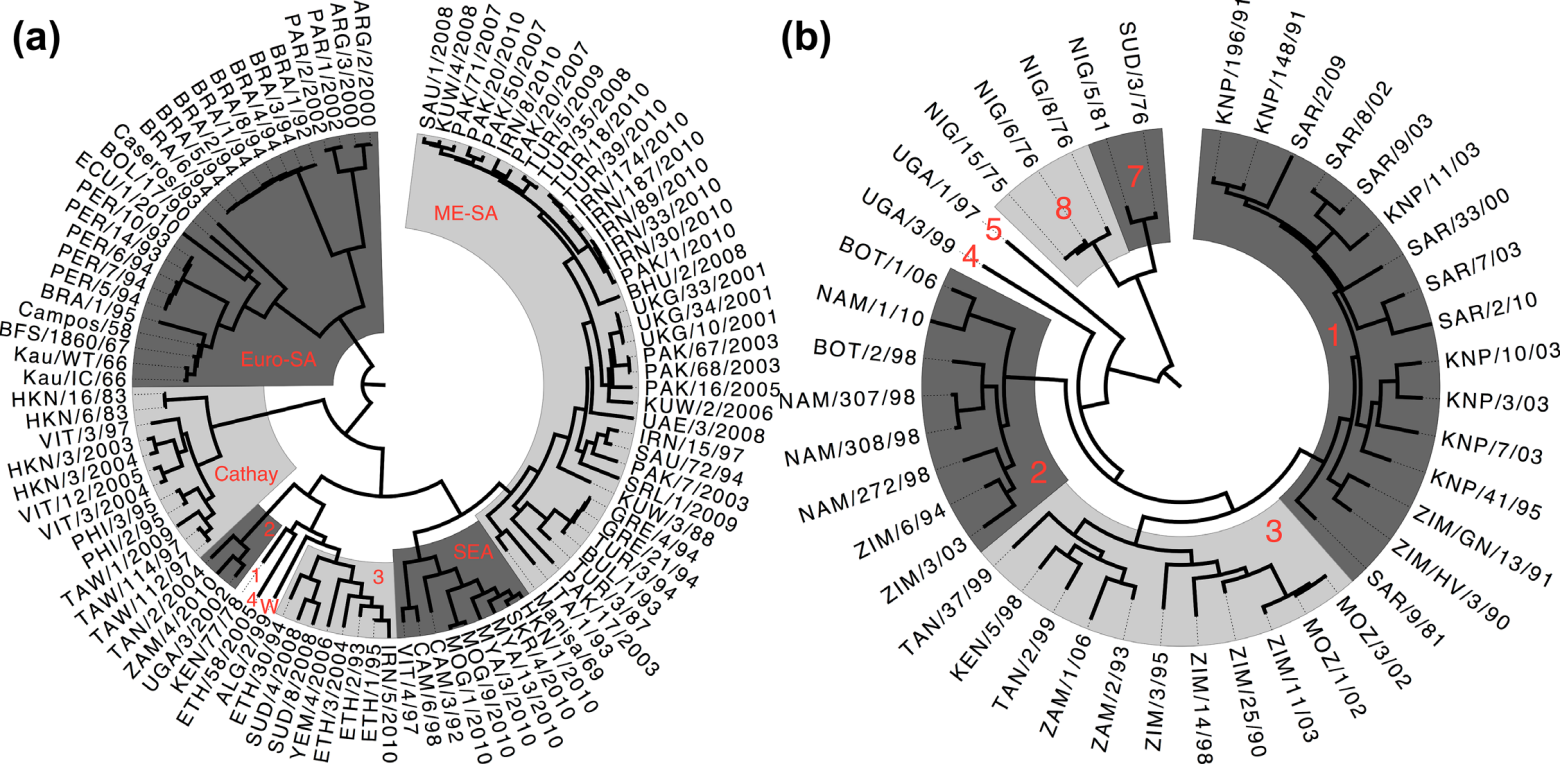
Phylogenetic tree for serotypes O and SAT1. (a) Phylogeny of all serotype O viruses studied, with alternate shading showing geographically-isolated subtypes (topotypes), labelled with their names in red (1, 2, 3 and 4 are East Africa topotypes EA-1, EA-2, EA-3 and EA-4, and W is the West Africa topotype, WA). The three unshaded viruses are all sole representatives of their topotype. (b) Phylogeny of all serotype SAT1 viruses studied (including those from[25]), with alternate shading showing topotypes, labelled with their numbers in red. The two unshaded viruses are sole representatives of their topotype. Phylogenies are time-resolved, with branch lengths measured in evolutionary time rather than substitutions.

Nucleotide sequences of the full capsid-coding regions were determined for the viruses selected for serology, where the sequences were not already available. Sequencing was performed on the same passage of the virus used for serology. Where viral stocks had previously been sequenced, less than 1% amino acid changes were observed, and these changes were not at any of the sites identified in the study. Amino acid sequence data for the structural proteins of all FMDV serotypes, including all of the O and SAT1 sequences used in this study, were deduced from nucleotide sequences and a common alignment was made for the VP1-4 proteins (S2 Table). Phylogenetic trees were then generated for each of serotypes O and SAT1 (Fig 1) from the nucleotide sequence data using a relaxed uncorrelated exponential clock [31], and a GTR+CP_112_+Г_112_+I nucleotide model [32].

### 2.3 Antigenic site analysis

To identify antigenic relationships and their predictors, we used linear mixed effects models [33] in R [34] to account for variation in logged pairwise cross-neutralization titres using viral sequences and structural data (see [25] and Materials and Methods). Experimental variability and other forms of non-antigenic variability frequently affect serological data. There is variability in the immune response against the same vaccine virus, which affects the antibody titre against all viruses. Initial model selection determined that test virus (p<10^−9^) and reference virus (p<10^−15^) should be included as fixed effects in the model–reflecting differences in the magnitude of titres when particular viruses or antisera are used irrespective of antigenic similarity. Run (technician on a specific date, p<10^−15^) and the individual animal serum (p<10^−15^) were controlled for as random effects–accounting for variability in the dilution of reagents, antisera and of virus isolates, and animal-to-animal variability in immunological response. Phylogenetic branches were then identified that were associated with drops in titre in order to control for repeated measures arising from phylogenetic correlations in the data. Finally, terms representing substitutions at each of the surface-exposed residues were tested to determine whether their inclusion significantly improved model fit [25]. This identified five residues with specific substitutions for serotype O VNT, five for serotype O LPBE (two shared between the assays) and eight for SAT1 VNT (Table 1).

**Table 1:** Residues identified by antigenic site analysis as forming part of FMDV epitopes.

Residue on common alignment	Serotypes where		Antigenically distinct amino acids^#^		
	residue	loop			
	is known to be antigenic		on O (VNT)	on O (LPBE)	on SAT1 (VNT)
**VP2 72** *^1^	O A C SAT1 Asia1	SAT2	.	.	D-E
**VP2 74** *^1^	O Asia1 C	A SAT1 SAT2	P-S^+^	.	.
**VP2 193**	O	A	S-T^+^	N-T^+^	.
**VP3 56** *^2^	O	A C Asia1	H-R	.	.
**VP3 72**	SAT1	A	.	.	N-S
**VP3 77**	SAT1	A	.	.	L-C^+^,L-R^+^
**VP3 138**	SAT1	A	.	.	D-K,E-K,R-K
**VP3 223**	Asia1		.	A-S-T	.
**VP1 47**	O		.	K-Q^+^	.
**VP1 142** *^3^		O A C SAT1 SAT2 Asia1	D-E-R	D-E^+^,R-E^+^	.
**VP1 144** *^3^	SAT1 C Asia1	O A SAT2	.	.	G-S,N-S
**VP1 149** *^3^	SAT1 A Asia1	O C SAT2	.	.	D-E-T
**VP1 164** *^3^	SAT2	O SAT1 A C Asia1	.	.	E-G^+^,E-S^+^
**VP1 169** *^3^		O SAT1 SAT2 A C Asia1	A-T	.	.
**VP1 209** *^4^		O A C SAT2	.	.	G-N-Q, A-Q^+^
**VP1 211** *^4^	O A C	SAT2	^^+^	D-E^+^	.

^*1-*4^ Part of one of four surface-exposed regions previously identified as antigenic on at least four serotypes of FMDV. Serotypes are recorded where those exact residues or nearby residues on the same β-loop are known to form part of epitopes from this or previous studies.

^#^Residues identified as antigenically important in a univariate analysis (p < 0.05) are shown in columns 4–6, identified according to their position in our common alignment (column 1). Residues are only shown where specific substitutions were also identified in the subsequent analysis as being associated with a drop of over 10% in titre. Antigenically distinct amino acids are shown separated by -.

^+^Not significant after Holm-Bonferroni correction.

^Antigenically significant residue, but no individual substitution was identified.

**Fig 2:**
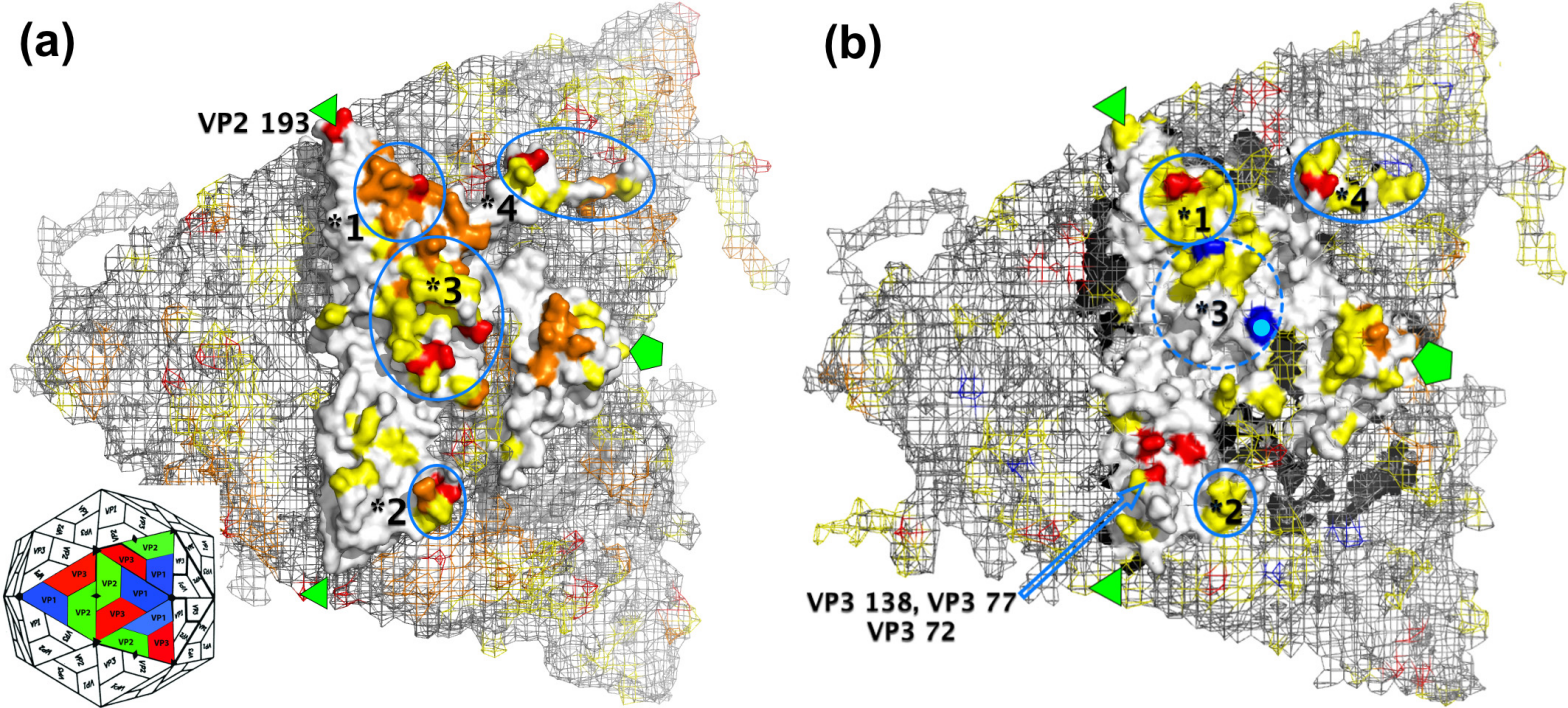
Antigenic sites on serotypes O and SAT1. The molecular surface of (a) serotype O and (b) serotype SAT1 icosahedral protomers is shown using pymol (Schrödinger LLC), with the molecular surface of neighbouring protomers shown as a light grey mesh (refer to inset icosahedral representation of an FMDV capsid for orientation). The molecular surface is white, with known antigenic sites on this serotype in orange (red when they were identified in this study), and known antigenic sites for any other serotype in yellow (see Table 1 and S3 Table for details). The location of the residues on the VP1 βG-βH loop could not be resolved on the serotype SAT1 structure, and so sites on that loop are not shown. Blue highlights mark the beginning (VP1 139) and end (VP1 165, with pale dot) of the disordered area, and the approximate locations of all four surface-exposed regions identified in Table 1 are also circled in blue and marked as before with *1-*4. Residues identified in this study but not in these regions are individually labelled with their positions on the common alignment. The three-fold and five-fold axes of symmetry are shown by triangles and pentagons respectively.

Sixteen residue positions with associated substitutions (Table 1 and shown on structures in Fig 2) are identified as potentially antigenically important across both serotypes (p < 0.05), ten of which are on the four surface-exposed regions identified as containing antigenic sites on at least four serotypes (Table 1, ^*1-*4^, and see S3 Table for further details). Five of the sixteen residue positions were located within and neighbouring the VP1 βG-βH loop (analysis of the SAT1 BOT/1/68 and O BFS/1860/67 structures shows that these residues form part of a single contiguous surface-exposed region of the capsid), which is the major antigenic site for FMDV [35] (Table 1, ^*3^). Of the sixteen, nine were significant after Holm-Bonferroni correction for the number of tests [36].

For serotype SAT1, five residues were identified as significant, all of which were validated from SAT1 mar-mutants (section 2.4, and Table 1, column 2, **SAT1**). Three further residues were identified as potentially important (p < 0.05) with identifiable antigenically important substitutions. These three residues are VP3 77, which was validated by another SAT1 mar-mutant; VP1 164, which is part of the VP1 βG-βH loop, a known epitope for serotypes A and C; and VP1 209, which is part of the VP1 C-terminus, which contains a known antigenic site on at least four other serotypes. The former five residues whose locations can be resolved on the serotype SAT1 structure are shown in red in Fig 2B.

For serotype O, four residues were identified as significant (Table 1, columns 3–4), of which three are part of structural loops that are known to contain antigenic sites on serotype O (Table 1, column 2, O). VP3 56 is part of a known epitope for four serotypes including serotype O, though the H56R substitution is known to be a cell-culture adaptation modification, which occurred during the passage of the TUR/5/2009 vaccine strain from the field isolate used in this study, which was not accounted for in the model. VP1 169 is part of a known epitope for serotypes A and C, while the VP1 142 is part of the VP1 βG-βH loop. The fourth residue, VP3 223 (identified by LPBE only), is located on the C-terminus of VP3, and has only previously been identified as antigenically important on serotype Asia 1 [37]. We therefore validated the VP3 T223A substitution identified by the model using site-directed mutagenesis (section 2.5). None of the other residues identified are known to be associated with cell culture in vitro [38–40].

Four further residues were identified as potentially important with identifiable substitutions, though with reduced statistical confidence: VP2 74 and VP1 47 are part of known epitopes on serotype O, and VP2 193 and VP1 211 are on loops containing epitopes for the serotype (Table 1). However, for VP2 193, different substitutions are identified for LPBE and VNT, suggesting that the assays are behaving differently, and contemporaneous work published separately by the authors [41] also investigated the VP2 T193N substitution in an engineered virus with a very unusual antigenic phenotype (it had all of the five known FMDV antigenic sites changed). The results of that study showed an effect at LPBE as predicted here, but also detected a small effect at VNT, however the statistical significance of those results was not analysed in that study. To investigate whether our new model was correct in finding that this substitution did not affect neutralisation of naturally circulating serotype O viruses, the VP2 T193N substitution identified for LPBE was also tested by site-directed mutagenesis in two viruses. All of these residues are shown on the serotype O structure along with other FMDV epitopes (Fig 2A).

The two substitutions chosen for testing were therefore: (a) at residues never previously identified by mAb escape mutant studies for serotype O; and (b) residues where the effect on VNT and LPBE was predicted to differ.

### 2.4 Serotype O site-directed mutagenesis and serology

Two residues were selected for mutagenesis, with substitutions at VP3 T223A (VP3 219 on the O BFS/1860/67 structure, Protein Data Bank ID: 1BBT [42]) and VP2 T193N (VP2 191 on structure). Both of these substitutions were selected by antigenic site analysis based on LPBE and not by VNT. Since the substitutions occurred within the evolutionary histories of the virus isolates used for both the LPBE and the VNT, this was not the result of the slightly different datasets used, and we predicted that both substitutions would cause a decrease in titre for LPBE but not for VNT. We also found a different substitution (VP2 T193S) to be important for VNT and not LPBE, but since that substitution did not occur in the evolutionary history of the isolates used for LPBE, it was not possible to predict its effect for LPBE. The VP3 223 residue is a known antigenic site for serotype Asia 1 (VP3 218 in [37]), but not for serotype O, and the VP2 193 residue is situated 7Å from known antigenic site VP2 190 (VP2 188 in [43]). The VP2 193 residue was also identified by an independent structure-based analysis [44].

We generated both the VP3 T223A and VP2 T193N mutations in an infectious O genome-length clone (O UKG/34/2001) to investigate (1) the antigenic analysis itself, and (2) the conclusions of that analysis, namely that there are qualitative differences between the LPBE and VNT assays. The latter differences are observed despite the two tests often being used interchangeably for measuring antigenic match between FMD viruses. To further investigate the VP2 T193N substitution, and to confirm that previous results [41] were atypical due to the unusual antigenic phenotype of the parental virus, we obtained a second engineered virus with an O Kau/WT/66 capsid (rO1K, [41] and Section 4.6) and an equivalent VP2 T193N substitution in the O Kau/WT/66 capsid.

The O UKG/34/2001 parental virus and the two, engineered, mutant viruses were tested by VNT against the O UKG/34/2001 antiserum used in the initial serological testing, and the O1K wild type and its mutant virus were also tested by VNT against an antiserum raised against the antigenically similar O BFS/1860/67 virus (Table 2). The O1K wild-type virus was antigenically more similar to BFS/1860/67 than to the Kau/IC/66 or the UKG/34/2001 virus, as seen from the higher neutralization titres against the BFS/1860/67 antiserum. For VNT none of the mutant viruses were significantly different from the parental viruses. This validates the predictions that the VP3 T223A and VP2 T193N substitutions are not antigenically significant for VNT.

**Table 2:** Antigenic impact of introduced substitutions on VNT and LPBE titres.

Substitution and background		Assay	Change caused by substitutions in log_2_ titre (±SE) for antisera raised against the following viruses		
			O UKG/34/2001	O BFS/1860/67	O Kau/IC/66
VP2 T193N	UKG^‡^	**LPBE**	-1.15 (±0.15)^*^	**-1.21 (±0.18)**	**-0.40 (±0.19)**
	UKG	VNT	+0.09 (±0.19)	ND^†^	ND
	rO1K	VNT	ND	0.10 (±0.18)	ND
VP3 T223A	UKG	**LPBE**	**-0.75 (±0.14)**	-0.02 (±0.18)	-0.04 (±0.17)
	UKG	VNT	0.01 (±0.19)	ND	ND

^*^Significant effects are shown in **bold**.

^†^ND = Not Done.

^‡^O UKG/34/2001.

In LPBE, the antigenic impact of the substitutions at VP2 193 (T-N) and VP3 223 (T-A) were tested on O UKG/34/2001 using three antisera: antiserum derived from the parental O UKG/34/2001, from a less antigenically similar virus (O BFS/1860/67), and from an antigenically very distant virus (O Kau/IC/66). Changes to both antigenic phenotypes were significant (Table 2, p<10^−15^). The drop in serum titre was larger for the VP2 193 T-N substitution, with an observed drop in titre of 0.40–1.21 antigenic units (the observed difference in log_2_ serum titre observed in either the VNT or LPBE assay) compared to the parental O UKG/34/2001 virus, with a significant (though reduced) effect even on the most distantly related virus. Similarly, a drop of up to 0.75 antigenic units was observed for the VP3 223 T-A substitution, although this smaller effect was not observed with antisera raised against more antigenically distinct viruses. Both mutant viruses were therefore significantly different from their parental viruses at LPBE, validating the predictions that both substitutions are antigenically significant for LPBE.

### 2.5 SAT1 epitope mapping using mar-mutants

Antibody binding sites on a SAT1 capsid were identified by characterizing viruses escaping neutralization by mAbs *in vitro* [20]. Twenty SAT1 BOT/1/68-specific mAbs were found to neutralize the infectivity of the homologous virus *in vitro*. Of these, eight strongly neutralizing mAbs were used to derive neutralization escape mutants from the parental SAT1 virus (3–5 independent mutation events for each mAb). Seventeen distinct mar-mutants were generated (see S4 Table and [20]), of which fourteen differed at one of the sites identified by antigenic modelling of serotype SAT1 (see Table 1, **SAT1** labels for matching sites). For the remaining three mutants, two had substitutions in common, so at least two further distinct antigenic sites were required to explain their resistance to neutralisation. Overall, out of fourteen residues with substitutions across all of the mar-mutants, six matched those in our antigenic site analysis conducted in Section 2.3 (Table 1). Although further cattle experiments would have been preferable to validate the antigenic site analysis, our ability to take advantage on existing work with murine antibodies greatly reduced the animal work necessary to validate the SAT1 results. While it was not clear from the serotype O work that we should necessarily expect a direct correspondence between cattle and mouse results, the correspondence we did observe with the SAT1 data shows that the technique does choose antigenic sites, and that these are the same for both species (Fisher’s exact test, p < 10^−7^ for all 14 sites, p < 10^−9^ for the minimal 8 sites required to explain resistance to neutralisation).

### 2.6 Antigenic evolution

**Fig 3:**
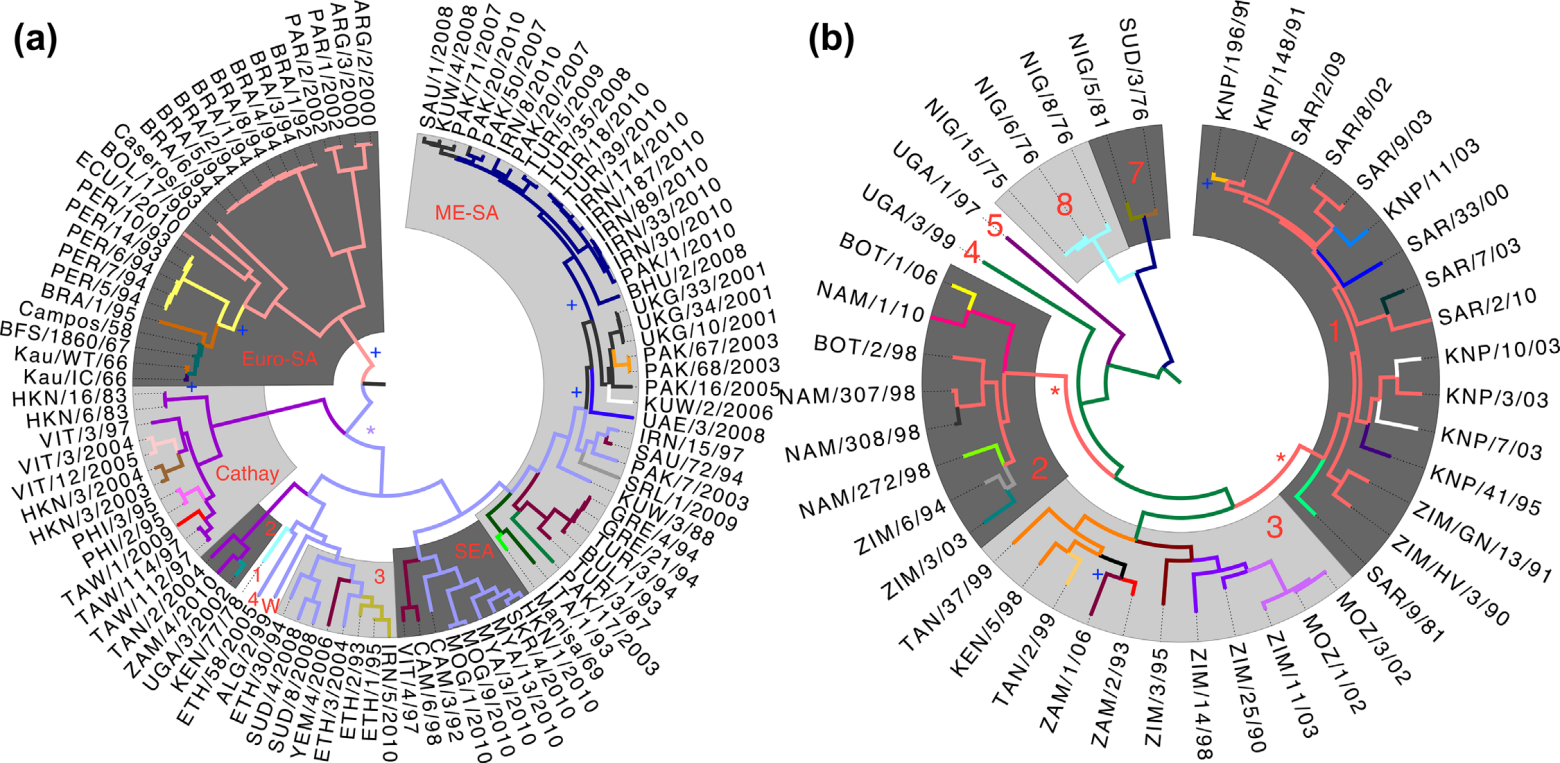
Antigenic evolution of Serotypes O and SAT1. Phylogeny of all serotype O viruses (a) and serotype SAT1 (b) are shown with shading and labelling of topotypes as Fig 1A–1B. Different branch colours denote antigenic dissimilarity with respect to VNT between the viruses derived from analyses of the sequence and serological data and ancestral state reconstruction. Conversely, the same colours in the same phylogeny denote the same antigenic phenotype. ^+^Change in antigenic phenotype for which no causative substitution has been identified. The broadest such group defined by VNT is starred for serotype O (spanning 5 topotypes) and SAT1 (spanning 2 topotypes). As in Fig 1, phylogenies are time-resolved, with branch lengths measured in evolutionary time rather than substitutions.

Ancestral state reconstruction was used to determine amino acid identity at each internal node of the phylogeny, revealing the pattern of substitutions of the residues identified as antigenically important for serotype O and SAT1 with respect to VNT (Fig 3, colours change along branches identified as containing antigenically important substitutions). As there is an overlap of just two residues between the O VNT and O LPBE results, a very different pattern of antigenic change is apparent between the two trees (Fig 3A and 3B respectively). However, we do see one point of agreement in that the viruses forming their own topotypes EA-4, WA and some viruses in EA-3 (labelled 4, W and 3, respectively) are neither evolutionarily distinct (having a common ancestor approximately 75 years ago) nor antigenically distinct, having a common ancestor of the same antigenic phenotype (colour). These topotypes are also phenotypically similar to viruses in SEA and ME-SA in VNT analysis. For SAT1, on the other hand, we see the topotypes are more distinct from both an antigenic and evolutionary perspective.

## 3. Conclusions

This study traced the antigenic history of two serotypes of FMDV using a novel analytical approach based on the synthesis of genetic, serological and structural data, simultaneously validated in a study on human influenza A (H1N1) [45]. In contrast to our previous studies, where we were only able to definitively identify broad antigenic regions of the capsid, here we identified nine specific residues with naturally occurring, antigenically important substitutions, all of which were either located on structural elements known from mar-mutant studies to contain epitopes for that serotype or were validated using site-directed mutagenesis.

Our new understanding of how the virus has evolved phenotypically since its separation into different serotypes makes it clear that there is little pressure to constantly evolve to escape immune pressure in this system, since we see antigenic phenotypes preserved across the whole phylogeny of both serotypes, and instead the virus has undergone a genetic and phenotypic diversification. This is in contrast, for example, to the findings of a study of the antigenic evolution of the trunk lineage in human influenza A (H3N2) [23]. We can also use these results to query the significance of the traditional division of FMD viruses into some topotypes. In the case of the East and West African topotypes EA-3, EA-4 and WA of serotype O in particular, not only do the viruses occur in overlapping geographical regions, they also have recent common ancestry and similar antigenic phenotypes.

The ability to reconstruct the phenotypic evolution of the virus in terms of specific substitutions also allows us to begin to predict the potential implications of future genotypic changes. While we caution that this analysis is certainly not in sufficient depth to have identified all of the antigenic sites for the two serotypes, as evidenced from the number of sites mapped by antigenic analysis for O and SAT1 and the total number of sites across the six serotypes identified by mar-mutant studies (81, S3 Table). However, approximately a third (27 and 22, respectively) of the residues identified as antigenically important by mar-mutant studies are not even naturally variable in amino acid alignments for the two serotypes we have studied, so 81 is likely to be a large overestimate of the number of viable substitution sites which are antigenically relevant for any given serotype. Our previous study was able to predict the cross-reactivity of strains with antisera through correlations between substitutions on surface-exposed regions and change in VNT titres [25], but our new ability to identify the specific causative substitutions, once expanded in terms of substitutions through further laboratory work, offers the potential to predict antigenic phenotype directly from sequence. Recombination may have occurred in the capsid during the evolutionary history of each of the two serotypes, affecting the phylogenetic reconstruction, but the geographical separation of the different topotypes makes it unlikely that this occurred across the topotypes, and the conclusions that the sites identified are antigenically important are in any event independently validated. In support of this view, Jackson *et al*. [46] showed that recombination is more likely to include the complete outer capsid-coding region rather than within the capsid-coding region.

The development of better vaccines is the critical pragmatic purpose underlying the analysis of the mechanistic basis for the evolution of antigenic phenotypes. Vaccinology, particularly for diseases like FMD and human influenza that are variable in their antigenic character, remains a conspicuously empirical science. It is rightly grounded in the sampling of novel field isolates and (currently) in *ex vivo* approximations to determine vaccine match. However, through better understanding of the molecular basis of antigenic evolution, we have already identified a potentially serious problem in the assays currently used for analysis of FMDV antigenic match. It has long been known that the correlation between LPBE and VNT is inadequate, but our results show that some substitutions are only relevant to LPBE and not to neutralisation tests based on cattle antisera. At least in part, therefore, different sites on the virus are “antigenic” for different assays. There were only three common antisera between the two datasets, making such a determination uncertain, but the specific substitutions identified were present in both. While it is therefore possible that the assays are still broadly overlapping in their antigenic sites, though our results do not suggest this, reverse genetics shows that LPBE is certainly identifying sites not observed with VNT. These may either be non-neutralising sites that may or may not be relevant for protection *in vivo* [47], or even antigenic sites relevant only to a different species (as the reagents used in the LPBE include rabbit and guinea pig antibodies). However, the strong correspondence between our SAT1 results in cattle and the murine mar-mutant results, previously recorded, suggests that species differences may not be the only reason. Whatever the underlying reason for the differences between the assays, the ultimate goal should be to go beyond simple serological ideas of antigenic match, and instead to make an explicit connection between heterologous *in vivo* protection and the *ex vivo* assays used to quantify antigenic similarity in order to move to a true *in vivo* antigenic site analysis. Either this will require likely impractical resources for extensive *in vivo* cross-protection studies or, perhaps more plausibly, more resources dedicated to outbreak monitoring and serosurveillance in endemic countries, where natural challenge experiments regularly occur.

## 4. Methods

### 4.1 Cells and viruses

One hundred and three serotype O viruses included in this study were obtained from the World Reference Laboratory (WRL) for FMD at the Pirbright Institute, Pirbright (United Kingdom). These viruses were selected to represent the broadest geographical distribution (S1 Table) and therefore to provide a wide range of antigenic relationships. The 25 SAT1 FMDV isolates from 8 countries in southern Africa (S1 Table) were supplied by the WRL for FMD at the Pirbright Institute or form part of the virus bank at the Transboundary Animal Diseases Programme (TADP) of the ARC-Onderstepoort Veterinary Institute (ARC-OVI, South Africa). The viruses were grown on IBRS-2 cells (a pig kidney cell line maintained at the Pirbright laboratory central service unit and at the Transboundary Animal Diseases Programme of ARC-OVI) to a titre of >4.5 log_10_ / ml (3–7 passages) and stored at -70°C until used. Two older reference strains were subsequently grown on BHK-21 clone 13 (strain 21; American Tissue Culture Collection CCL-10) cells (a baby hamster kidney cell line maintained at the Pirbright laboratory central service unit and at the Transboundary Animal Diseases Programme of ARC-OVI). IBRS-2 and BHK-21 clone 13 cells were used for virus titration and electroporation to recover virus from cDNA clones, respectively. Recombinant viruses were grown either on BHK-21 or goat tongue epithelial cells. IBRS-2 cells were used to grow mutant viruses resistant to neutralization by SAT1 mAbs.

### 4.2 RNA extraction, RT- PCR and nucleotide sequencing

Total RNA from cell culture grown viruses was extracted using the RNeasy kit (Qiagen) as per the manufacturer’s instructions. For serotype O viruses reverse transcription (RT), polymerase chain reaction (PCR) to amplify capsid encoding genes [using primer pair L463F (ACCTCCRACGGGTGGTACGC)/NK72R (GAAGGGCCCAGGGTTGGACTC) or L463F/EUR2B52R (GACATGTCCTCCTGCATCTGGTTGAT)] and nucleotide sequencing were performed as described previously [48]. Additional serotype O specific sequencing primers were used to sequence the capsid on both the strands. For serotype SAT1 viruses, RT-PCR to amplify capsid encoding genes and nucleotide sequencing were performed as described previously [25]. Capsid (P1) nucleotide sequences of 103 serotype O viruses and 43 SAT1 viruses were aligned using the ClustalW v2.1 multiple sequence alignment program to create a common cross-serotype alignment. This provides a common naming scheme for comparison of epitopes between the serotypes for which there is existing mAb escape mutant data, the antigenic analyses were carried out on serotype O and SAT1, and the site-directed mutagenesis work was carried out on serotype O. The GenBank accession numbers of the capsid-coding sequences are shown in S1 Table.

### 4.3 Preparation of bovine antiserum and serological tests

Five serotype O post-vaccination bovine antisera (O Kau/IC/66, O BFS/1860/67, O UKG/34/2001, O KEN/77/78 and O UGA/3/2002) were obtained, which had been previously generated at the isolation unit of the Pirbright Institute by vaccinating 5 cattle with 10 micrograms of respective vaccine antigen per dose after sublimating with montanide ISA 206 (Sepic, France). Two antisera (O Manisa/69 and O TUR/5/2009) were procured from Intervet, Germany. These antisera were all of those available for which full capsid sequence data of the seed strains could be obtained. These were either 21-day or 28-day post-vaccination antisera and pooled from five animals in each vaccine test. The strain differentiation liquid phase blocking ELISA (LPBE) was carried out essentially as described by previously [30]. Two-dimensional virus neutralisation tests were also carried out following established methodology [29]. Antibody titres were calculated from regression data as the log_10_ reciprocal antibody dilution required for 50% neutralisation of 100 tissue culture infective units of virus (log_10_SN_50_/100 TCID_50_).

Serotype SAT1 convalescent sera were obtained from cattle 28 days post-infection with the reference viruses SAT1 KNP/196/91, SAT1 SAR/9/81, SAT1 BOT/1/06 and SAT1 ZAM/1/06. These strains were chosen as antigenically representative of three of the topotypes studied previously [25]. For this purpose, a group of five cattle were inoculated intradermolingually with 10^4^ TCID_50_ of each virus. The antigenic cross-reactivity of SAT1 viruses against the test sera were determined using a virus neutralization test as described above. All the serological tests were repeated on different dates between one and five times.

### 4.4 Phylogenetic analysis and ancestral state reconstruction

Bayesian estimation of phylogeny was generated from aligned P1 nucleotide sequences with a variety of nucleotide substitution and molecular clock models being tested through calculation of Bayes Factors [49]. Phylogeny construction and analysis was carried out using BEAST v1.7.2 and Tracer v1.5 [50]. The pattern of evolutionary history at each of the sites identified during statistical modelling was revealed by reconstruction of amino acid state at all hypothetical ancestors of a phylogeny generated with an identical nucleotide model, using the blosum62 [51] model of amino acid substitution and a an unlinked strict molecular clock [52] for each antigenic residue. These reconstructed amino acid states were used to identify the branches of the phylogeny that carry substitutions between amino acids determined to be antigenically distinct (Table 1). These branches are indicated by colour changes in Fig 3.

### 4.5 Structural analysis and determination of candidate regions

The crystal structures of O BFS/1860/67 ([53], PDB ID: 1BBT) and SAT1 BOT/1/68 ([25], PDB ID: 2WZR) were used to prepare a multimeric structure. The molecular surface was inspected using the PyMol Molecular Graphics System v1.2r0 (DeLano Scientific LLC) and surface exposed residues were identified, and the structure was also analysed using the PISA software in order to determine the surface area of amino acids already involved in interactions with surrounding amino acids and therefore not available for interaction with antibody.

### 4.6 Construction of plasmids, site directed mutagenesis and recovery of mutant viruses

FMDV cDNA plasmid pT7S3-O1K/O UKG35 [54] was used in this study. This plasmid is the O1K cDNA clone where the capsid-coding region is substituted with the equivalent capsid coding region of O UKG/35/2001 virus. However, prior to the substitution of these residues an earlier substitution was made to change VP3 56 from histidine (H) to arginine (R) to match O UKG/34/2001, which was one of the vaccine strains used for both the VNT and LPBE work. This ensured that the backbone from the chimeric virus was identical to the original virus that was used to generate the serological data. A standard site-directed mutagenesis technique was used to introduce point mutations at specific positions in the capsid (S2 Table). *In vitro* transcribed RNA was electroporated into BHK-21 cells essentially as described previously [55] and the recovered viruses were grown using either IBRS-2 cell line or goat tongue epithelial cells. The identities of the recovered viruses were confirmed by RT-PCR followed by sequencing. In order to study the effect of these residues infectious viruses were recovered from at least two different clones of the two mutant and wild-type plasmids. Sequencing confirmed that all the mutant viruses retained the mutation up to the third passage. The viruses were also tested to ensure that they grew to a similar titre as the parent virus and that the phenotypes of the viruses on cell culture were similar. The single mutant virus, rO1K-VP2-191 that contains a T-N substitution at VP2-191 position in O1K-wt capsid [41] was also used in this study.

### 4.7 Data, Statistical modelling and model selection

All statistical analyses were carried out using R v3.0.1 [34] and mixed effects models using the lme4 package [33]. Similar techniques to those used here are now published [45], but abbreviated versions are provided here to clarify the small differences in the analyses. Antibody titres (log_2_(*t*_*r*,*v*_)) were used as the response variable throughout as their residuals in the models were homoscedastic. Goodness of fit of models including each of the following variables was assessed by likelihood ratio test: the avidity of the test virus, *a*_*v*_, and the immunogenicity of the reference virus, *g*_*r*,_ against which the antiserum was raised (the vaccine seed strain or the infection strain) as fixed effects, and the same terms and the date of test, *ε*_*D*_, as a random effect for both models, as well as fixed effects for technician and individual animal serum, and random effects for individual animal serum, *ε*_*s*_, and the run, *ε*_*R*_ – technician on day of test to distinguish multiple technicians on the same date–for the serotype SAT1 model, where multiple technicians and individual antisera were used in testing. *a*_*v*_, *g*_*r*,_ and *ε*_*D*_ were found to be significant (p<0.05) after correcting for multiple testing [36] for serotype O, and *a*_*v*_, *g*_*r*,_ *ε*_*S*_ and *ε*_*R*_ for serotype SAT1, making the models consistent within the constraints of the slight differences in experimental design. To prevent false support for substitutions due to repeated measurements, branches of the O and SAT1 phylogenies containing antigenicity changing events were identified and added to the model (as Eq 2 of [45]), generating the following equations for serotype O and SAT1 respectively:

$${\text{log}}_{2}\left(t_{r,v}\right)\;=\;k_{0}+\sum_{i}m_{i}\delta_{i}\left(r,v\right)+g_{r}+a_{v}+\varepsilon_{D}+\varepsilon$$ (1)

$${\text{log}}_{2}\left(t_{r,v}\right)\;=\;k_{0}+\sum_{i}m_{i}\delta_{i}\left(r,v\right)+g_{r}+a_{v}+\varepsilon_{s}+\varepsilon_{R}+\varepsilon$$ (2)

where *k*_*0*_ is the intercept and *ε* is the residual error. Eqs 1 and 2 incorporate branch terms through *m*_*i*_*δ*_*i*_(*r*,*v*), where *δ*_*i*_ = 1 when the reference strain (*r*) and test virus (*v*) are separated by branch *i* of the phylogeny and *δ*_*i*_ = 0 otherwise, with *m*_*i*_ being the associated regression coefficient from the mixed effects model. Because of the size of the search space, random restart hill-climbing was used to determine the best model [56]. To a random consistent starting model, branch terms were added and removed at random to maximize model fit, assessed by AIC [57]. This was repeated while randomizing their order to identify the best model to avoid sensitivity to the order in which the parameters were presented. This approach was conservative since it was used to determine the branches used to control for phylogenetic correlations in the data, and adding in extra unnecessary terms simply reduced the power of the analysis. The model was then extended with an explicit term for amino acid substitutions (as Eq 3 of [45]), to identify antigenically important residues:

$${\text{log}}_{2}\left(t_{r,v}\right)\;=\;k_{0}+k_{1}{\text{d}}_{1}\left(r,v\right)+\sum_{i}m_{i}\delta_{i}\left(r,v\right)+g_{r}+a_{v}+\varepsilon_{D}+\varepsilon$$ (3)

$${\text{log}}_{2}\left(t_{r,v}\right)\;=\;k_{0}+k_{1}{\text{d}}_{1}\left(r,v\right)+\sum_{i}m_{i}\delta_{i}\left(r,v\right)+g_{r}+a_{v}+\varepsilon_{s}+\varepsilon_{R}+\varepsilon$$ (4)

where *d*_1_ is the count of substitutions between *r* and *v* at a specific site, and *k*_1_ the associated regression coefficient. We used the resultant model to identify individual positions at which substitutions were significantly associated (p < 0.05) with a drop in cross-reactivity both before and after correcting for multiple testing (Table 1) [36]. By controlling for repeated measures, branch terms in the model remove significant direct effects of substitutions in individual branches, and inclusion of residue terms only improves goodness of fit when there is independent support across the phylogeny: *i*.*e*. convergent, alternative or back-substitutions at the same position associated with a drop in cross-reactivity in at least two branches [25].

A fixed effect was then incorporated instead of *k*_1_d_1_(*r*,*v*) for each residue identified as antigenically important (p < 0.05), with levels for every substitution observed between two reference viruses (appropriate subsets of the data were taken so that only substitutions observed between reference viruses were tested, as they could be unambiguously identified as antigenic, rather than causing an increase or decrease in overall reactivity of the virus with antisera). Identified substitutions that caused a drop of more than 10% in titre are presented in Table 1. All of the identified substitutions are then added into a single equation, and the branches of the O and SAT1 phylogenies containing the remaining unexplained antigenicity changing events were identified as before and added to the model:

$${\text{log}}_{2}\left(t_{r,v}\right)\;=\;k_{0}+\sum_{j}k_{j}\alpha_{j}\left(r,v\right)+\sum_{i}m_{i}\delta_{i}\left(r,v\right)+g_{r}+a_{v}+\varepsilon_{D}+\varepsilon$$ (5)

$${\text{log}}_{2}\left(t_{r,v}\right)\;=\;k_{0}+\sum_{j}k_{j}\alpha_{j}\left(r,v\right)+\sum_{i}m_{i}\delta_{i}\left(r,v\right)+g_{r}+a_{v}+\varepsilon_{s}+\varepsilon_{R}+\varepsilon$$ (6)

Ancestral state reconstruction was used to identify branches where substitutions causing a drop of over 10% occurred, and other branches in the model whose regression coefficients were over this size and therefore are assumed to contain unidentified antigenic changes of the same size (starred), are displayed visually in Fig 3 as colour changes in the phylogeny.

### 4.8 Ethics Statement

Cattle were housed in the bio-safety level 3 laboratories at TADP and all procedures were approved by the ARC-OVI Animal Ethics Committee (protocol numbers AEC 4.09 and AEC 34.09) and performed with the permission of the Department of Agriculture, Forestry and Fisheries (DAFF), South Africa (permit number 12/11/1/1). All animal work was performed adhering to the following national animal welfare standards: Animal Protection Act, 1962 (Act no 71 of 1962), Societies for the Prevention of Cruelty to Animals Act, 1993 (Act no. 169 of 1993), Veterinary and Para-Veterinary Professions Act, 1982 (Act no 19 of 1982), and the South African National Standard–“The care and use of animals for scientific purposes” SANS 10386.

## Supporting Information

S1 Data VNT serological results for serotype O viruses and antisera. (CSV)

S2 Data LPBE serological results for serotype O viruses and antisera. (CSV)

S3 Data VNT serological results for serotype SAT1 viruses and antisera. (CSV)

S1 Table Foot-and-mouth disease virus details with accession numbers. (DOCX)

S2 Table Pan-serotypic reference alignment of FMDV. The dataset shows the aligned VP2, VP3 and VP1 proteins of example SAT1 and O isolates used in the study alongside representative isolates from the other five serotypes. The four contiguous surface-exposed structural motifs confirmed as containing antigenic sites on at least four serotypes are highlighted in red–locations are approximate due to structural differences between the serotypes. The RGD cell surface receptor-binding motif, in the centre of the third site, is highlighted in blue.
(DOCX)

S3 Table Residues identified as part of epitopes on structural proteins across the six tested serotypes of FMDV, along with corresponding positions on all serotypes. (PDF)

S4 Table SAT1 mar-mutants. (DOCX)
