# Supplementary material for: Tracking the Antigenic Evolution of Foot-and-Mouth Disease Virus
Source: PLoS One. 2016 Jul 22;11(7):e0159360. doi: 10.1371/journal.pone.0159360 (PMC4957747; doi:10.1371/journal.pone.0159360)
Supplement: S1 Table — (DOCX) [file pone.0159360.s004.docx]

Table S1: Foot-and-mouth disease virus details with accession numbers

| Serotype | Virus | Topotype | Species | Passage history | Year isolated | Country  of origin | Genbank accession number |
| --- | --- | --- | --- | --- | --- | --- | --- |
| O | ALG/2/99 | WA | Cattle | BTY1 RS3 | 1999 | Algeria | KJ831737 |
| O | ARG/2/2000 | EURO-SA | Cattle | BTY1 RS3 | 2000 | Argentina | KJ831738 |
| O | ARG/3/2000 | EURO-SA | Cattle | BTY1 RS3 | 2000 | Argentina | KJ831739 |
| O | BFS/1860/67* | EURO- SA | Cattle | BTY1 RS3 | 1967 | UK | AY593815 |
| O | BHU/2/2008 | ME-SA | Cattle | BTY1 RS3 | 2008 | Bhutan | KJ831740 |
| O | BOL/17/90 | EURO-SA | Cattle | BTY1 RS3 | 1990 | Bolivia | KJ831663 |
| O | Campos/BRA/58* | EURO-SA | Not known | BTY1 RS3 | 1958 | Brazil | AJ320488 |
| O | BRA/1/92 | EURO-SA | Cattle | BTY1 RS3 | 1992 | Brazil | KJ831741 |
| O | BRA/1/94 | EURO-SA | Cattle | BTY1 RS3 | 1994 | Brazil | KJ831742 |
| O | BRA/1/95 | EURO-SA | Cattle | BTY1 RS3 | 1995 | Brazil | KJ831748 |
| O | BRA/2/94 | EURO-SA | Cattle | BTY1 RS3 | 1994 | Brazil | KJ831743 |
| O | BRA/3/94 | EURO-SA | Cattle | BTY1 RS3 | 1994 | Brazil | KJ831744 |
| O | BRA/4/94 | EURO-SA | Cattle | BTY1 RS3 | 1994 | Brazil | KJ831736 |
| O | BRA/5/94 | EURO-SA | Cattle | BTY1 RS3 | 1994 | Brazil | KJ831745 |
| O | BRA/6/94 | EURO-SA | Cattle | BTY1 RS3 | 1994 | Brazil | KJ831746 |
| O | BRA/8/94 | EURO-SA | Cattle | BTY1 RS3 | 1994 | Brazil | KJ831747 |
| O | BUL/1/93 | ME-SA | Cattle | BTY1 RS3 | 1993 | Bulgaria | KJ831664 |
| O | CAM/3/92 | SEA | Cattle | BTY1 RS3 | 1992 | Cambodia | KJ831665 |
| O | CAM/6/98 | SEA | Pig | BTY1 RS3 | 1998 | Cambodia | KJ831666 |
| O | Caseros/ARG/93* | EURO-SA | Not known | BTY1 RS3 | 1993 | Argentina | U82271 |
| O | ECU/1/2010 | EURO-SA | Cattle | BTY1 RS1 | 2010 | Ecuador | KJ831672 |
| O | ETH/1/95 | EA-3 | Cattle | BTY1 RS3 | 1995 | Ethiopia | KJ831669 |
| O | ETH/2/93 | EA-3 | Cattle | BTY1 RS3 | 1993 | Ethiopia | KJ831667 |
| O | ETH/3/2004 | EA-3 | Cattle | BTY1 RS3 | 2004 | Ethiopia | KJ831670 |
| O | ETH/30/94 | EA-3 | Cattle | BTY1 RS3 | 1994 | Ethiopia | KJ831668 |
| O | ETH/58/2005 | EA-4 | Cattle | BTY1 RS3 | 2005 | Ethiopia | KJ831671 |
| O | GRE/21/94 | ME-SA | Cattle | BTY1 RS3 | 1994 | Greece | KJ831674 |
| O | GRE/4/94 | ME-SA | Cattle | BTY1 RS3 | 1994 | Greece | KJ831675 |
| O | HKN/3/2003 | CATHAY | Pig | BTY1 RS3 | 2003 | Hong Kong | KJ831677 |
| O | HKN/3/2004 | CATHAY | Pig | BTY1 RS3 | 2004 | Hong Kong | KJ415243 |
| O | HKN/6/83* | CATHAY | Pig | BTY1 RS3 | 1983 | Hong Kong | KJ831676 |
| O | HKN/1/2010 | SEA | Pig | BTY1 RS1 | 2010 | Hong Kong | KJ831683 |
| O | IRN/15/97 | ME-SA | Cattle | BTY1 RS3 | 1997 | Iran | KJ831684 |
| O | IRN/5/2010 | EA3 | Cattle | BTY1 RS1 | 2010 | Iran | KJ831685 |
| O | IRN/8/2010 | ME-SA | Cattle | BTY1 RS1 | 2010 | Iran | KJ831686 |
| O | IRN/30/2010 | ME-SA | Cattle | BTY1 RS1 | 2010 | Iran | KJ831687 |
| O | IRN/33/2010 | ME-SA | Cattle | BTY1 RS1 | 2010 | Iran | KJ831688 |
| O | IRN/89/2010 | ME-SA | Sheep | BTY1 RS1 | 2010 | Iran | KJ831689 |
| O | IRN/174/2010 | ME-SA | Sheep | BTY1 RS1 | 2010 | Iran | KJ831690 |
| O | IRN/187/2010 | ME-SA | Cattle | BTY1 RS1 | 2010 | Iran | KJ831691 |
| O | ITA/1/93 | ME-SA | Cattle | BTY1 RS3 | 1993 | Italy | KJ415244 |
| O | Kaufbeuren/FRG/66* | EURO-SA | Cattle | BTY1 BHK7 | 1966 | Germany | X00871 |
| O | KEN/77/78 | EA-1 | Cattle | BTY1 RS3 | 1978 | Kenya | KP202877 |
| O | KUW/2/2006 | ME-SA | Cattle | BTY1 RS3 | 2006 | Kuwait | KJ831693 |
| O | KUW/3/88 | ME-SA | Cattle | BTY1 RS3 | 1988 | Kuwait | KJ831692 |
| O | KUW/4/2008 | ME-SA | Cattle | BTY1 RS3 | 2008 | Kuwait | KJ831694 |
| O | Manisa/TUR/69* | ME-SA | Cattle | BTY1 RS3 | 1969 | Turkey | AJ251477 |
| O | MOG/1/2010 | SEA | Cattle | BTY1 RS3 | 2010 | Mongolia | KJ831695 |
| O | MOG/9/2010 | SEA | Mongolian gazelle | BTY1 RS3 | 2010 | Mongolia | KJ831696 |
| O | MYA/3/2010 | SEA | Cattle | BTY1 RS1 | 2010 | Myanmar | KJ831698 |
| O | MYA/13/2010 | SEA | Cattle | BTY1 RS1 | 1020 | Myanmar | KJ831697 |
| O | PAK/7/2003 | ME-SA | Cattle | BTY1 RS3 | 2003 | Pakistan | KJ831720 |
| O | PAK/16/2005 | ME-SA | Cattle | BTY1 RS3 | 2005 | Pakistan | KJ831724 |
| O | PAK/17/2003 | ME-SA | Cattle | BTY1 RS3 | 2003 | Pakistan | KJ831721 |
| 1 | PAK/20/2007 | ME-SA | Cattle | BTY1 RS3 | 2007 | Pakistan | KP720594 |
| O | PAK/50/2007 | ME-SA | Cattle | BTY1 RS3 | 2007 | Pakistan | KP720595 |
| O | PAK/67/2003 | ME-SA | Cattle | BTY1 RS3 | 2003 | Pakistan | KJ831722 |
| O | PAK/68/2003 | ME-SA | Cattle | BTY1 RS3 | 2003 | Pakistan | KJ831723 |
| O | PAK/71/2007 | ME-SA | Cattle | BTY1 RS3 | 2007 | Pakistan | KJ831725 |
| O | PAK/1/2010 | ME-SA | Cattle | BTY1 RS1 | 2010 | Pakistan | KJ831726 |
| O | PAK/20/2010 | ME-SA | Cattle | BTY1 RS1 | 2010 | Pakistan | KJ831727 |
| O | PAR/1/2002 | EURO-SA | Cattle | BTY1 RS3 | 2002 | Paraguay | KJ831728 |
| O | PAR/2/2002 | EURO-SA | Cattle | BTY1 RS3 | 2002 | Paraguay | KJ831729 |
| O | PER/10/93 | EURO-SA | Cattle | BTY1 RS3 | 1993 | Peru | KJ831730 |
| O | PER/14/93 | EURO-SA | Cattle | BTY1 RS3 | 1993 | Peru | KJ831731 |
| O | PER/5/94 | EURO-SA | Cattle | BTY1 RS3 | 1994 | Peru | KJ831732 |
| O | PER/6/94 | EURO-SA | Cattle | BTY1 RS3 | 1994 | Peru | KJ831733 |
| O | PER/7/94 | EURO-SA | Cattle | BTY1 RS3 | 1994 | Peru | KJ831734 |
| O | PHI/2/95 | CATHAY | Pig | BTY1 RS3 | 1995 | Philippines | KJ415245 |
| O | PHI/3/95 | CATHAY | Pig | BTY1 RS3 | 1995 | Philippines | KJ831735 |
| O | SAU/72/94 | ME-SA | Cattle | BTY1 RS3 | 1994 | Saudi Arabia | KJ831699 |
| O | SAU/1/2008 | ME-SA | Cattle | BTY1 RS3 | 2008 | Saudi Arabia | KJ831700 |
| O | SAU/1/2009 | ME-SA | Cattle | BTY1 RS3 | 2009 | Saudi Arabia | KJ831701 |
| O | SKR/4/2010 | SEA | Cattle | BTY1 RS3 | 2010 | South Korea | KJ831702 |
| O | SRL/1/2009 | ME-SA | Buffalo | BTY1-RS3 | 2009 | Sri Lanka | KJ831703 |
| O | SUD/4/2008 | EA-3 | Cattle | BTY1 RS3 | 2008 | Sudan | KJ831704 |
| O | SUD/8/2008 | EA-3 | Cattle | BTY1 RS3 | 2008 | Sudan | KJ831705 |
| O | TAW/112/97 | CATHAY | Pig | BTY1 RS3 | 1997 | Taiwan | KJ831707 |
| O | TAN/2/2004 | EA-2 | Cattle | BTY1 RS3 | 2004 | Tanzania | KJ831706 |
| O | TAW/114/97 | CATHAY | Pig | BTY1 RS3 | 1997 | Taiwan | KJ831708 |
| O | TAW/1/2009 | CATHAY | Pig | BTY1 RS3 | 2009 | Taiwan | KJ831709 |
| O | TUR/3/87 | ME-SA | Not known | BTY1 RS3 | 1987 | Turkey | KJ831710 |
| O | TUR/3/94 | ME-SA | Cattle | BTY1 RS3 | 1994 | Turkey | KJ831711 |
| O | TUR/35/2008 | ME-SA | Cattle | BTY1 RS3 | 2008 | Turkey | KJ831712 |
| O | TUR/5/2009 | ME-SA | Cattle | BTY1 RS3 | 2009 | Turkey | KP202878 |
| O | TUR/18/2010 | ME-SA | Cattle | BTY1 RS3 | 2010 | Turkey | KJ831713 |
| O | TUR/39/2010 | ME-SA | Cattle | BTY1 RS3 | 2010 | Turkey | KJ831714 |
| O | UAE/3/2008 | ME-SA | Sand Gazelle | BTY1 RS3 | 2008 | UAE | KJ831715 |
| O | UAE/4/2009 | ME-SA | Blackbuck | BTY1 RS3 | 2009 | UAE | KJ831716 |
| O | UGA/3/2002 | EA-2 | Not known | BTY1 RS3 | 2002 | Uganda | KJ415246 |
| O | UKG/10/2001* | ME-SA | Pig | BTY1 RS3 | 2001 | UK | AJ311722 |
| O | UKG/33/2001 | ME-SA | Pig | BTY1 RS3 | 2001 | UK | KJ831717 |
| O | UKG/34/2001 | ME-SA | Pig | BTY1 RS3 | 2001 | UK | KJ831678 |
| O | VIT/12/2005 | CATHAY | Pig | BTY1 RS3 | 2005 | Vietnam | KJ831718 |
| O | VIT/3/2004 | CATHAY | Pig | BTY1 RS3 | 2004 | Vietnam | KJ831681 |
| O | VIT/3/97 | CATHAY | Pig | BTY1 RS3 | 1997 | Vietnam | KJ831679 |
| O | VIT/4/97 | SEA | Cattle | BTY1 RS4 | 1997 | Vietnam | KJ831680 |
| O | YEM/4/2006 | EA-3 | Cattle | BTY1 RS3 | 2006 | Yemen | KJ831682 |
| O | ZAM/4/2010 | EA-2 | Cattle | BTY1 RS3 | 2010 | Zambia | KJ831719 |
| SAT1 | KNP/196/91 | 1 | Buffalo | PK1RS5 | 1991 | South Africa | AF283429 |
| SAT1 | KNP/3/03 | 1 | Buffalo | PK1RS5 | 2003 | South Africa | KJ999914 |
| SAT1 | KNP/7/03 | 1 | Buffalo | PK1RS4 | 2003 | South Africa | KJ999915 |
| SAT1 | KNP/10/03 | 1 | Buffalo | PK1RS6 | 2003 | South Africa | KJ999916 |
| SAT1 | KNP/11/03 | 1 | Buffalo | PK1RS6 | 2003 | South Africa | KJ999917 |
| SAT1 | SAR/9/81 | 1 | Impala | PK1RS4BHK5 | 1981 | South Africa | DQ009715 |
| SAT1 | SAR/33/00 | 1 | Cattle | PK1RS6 | 2000 | South Africa | KJ999908 |
| SAT1 | SAR/8/02 | 1 | Buffalo | PK2RS5 | 2002 | South Africa | KJ999909 |
| SAT1 | SAR/7/03 | 1 | Buffalo | PK1RS4 | 2003 | South Africa | KJ999910 |
| SAT1 | SAR/9/03 | 1 | Buffalo | PK2RS1 | 2003 | South Africa | KJ999911 |
| SAT1 | SAR/2/09 | 1 | Cattle | PK1RS1 | 2009 | South Africa | KJ999912 |
| SAT1 | SAR/2/10 | 1 | Buffalo | RS1 | 2010 | South Africa | KJ999913 |
| SAT1 | BOT/2/98 | 2 | Buffalo | BTY2RS3 | 1998 | Botswana | KJ999918 |
| SAT1 | BOT/1/06 | 2 | Cattle | PK1RS6 | 2006 | Botswana | KJ999919 |
| SAT1 | NAM/272/98 | 2 | Buffalo | PK1RS5 | 1998 | Namibia | KJ999921 |
| SAT1 | NAM/308/98 | 2 | Buffalo | PK1RS5 | 1998 | Namibia | KJ999922 |
| SAT1 | NAM/1/10 | 2 | Cattle | PK1RS4 | 2010 | Namibia | KJ999923 |
| SAT1 | ZIM/3/03 | 2 | Cattle | PK1RS5 | 2003 | Zimbabwe | KJ999926 |
| SAT1 | MAL/1/85 | 3 | Buffalo | BTY1RS5 | 1985 | Malawi | AF056509 |
| SAT1 | MOZ/1/02 | 3 | Cattle | PK1RS5 | 2002 | Mozambique | KJ999928 |
| SAT1 | TAN/2/99 | 3 | Cattle | BTY1RS5 | 1999 | Tanzania | KJ999931 |
| SAT1 | ZAM/2/93 | 3 | Buffalo | PK1RS4 | 1993 | Zambia | KJ999929 |
| SAT1 | ZAM/1/06 | 3 | Cattle | PK1RS5 | 2006 | Zambia | KJ999930 |
| SAT1 | ZIM/3/95 | 3 | Buffalo | BTY1RS4 | 1995 | Zimbabwe | KJ999924 |
| SAT1 | ZIM/14/98 | 3 | Buffalo | BTY2RS4 | 1998 | Zimbabwe | KJ999925 |
| SAT1 | ZIM/11/03 | 3 | Cattle | PK1RS7 | 2003 | Zimbabwe | KJ999927 |

* These serotype O viruses are longstanding reference strains which have been previously uploaded to Genbank, mostly full genome sequences. In many cases we use different names for them in our analyses, often for slightly different sequences. All changes are relative to the start of P1:

- BFS/1860/67 retains its name, but has sequence changes a1076g, a1088g.
- Campos/BRA/58 is referred to as Campos/58 with the same sequence.
- Caseros/ARG/93 is referred to as Caseros/93 and has sequence changes g3a, t323c, a390g, a501g, c882t, c885t, t900c, a915c, t930c, g932t, c933t, c936t, c948t, c1026g, g1076a, c1161g, g1162c, n1168t, t1267c, n1268g, g1567c, t2000a, t2001g, g2202a.
- HKN/6/83 originated from the same sample as HKN/16/83, which is therefore not submitted, but this has sequence change a640c.
- Kaufbeuren/FRG/66 is referred to as Kau/IC/66 (infectious copy) and Kau/WT/66 (wild type). The latter has a different sequence and passage history – the passage history is BTY1 RS3 and sequence changes t618g, a644g, g1076a, g1088a, c1112t, a1164c, g1276a, g1427a, g1966a.
- Manisa/TUR/69 is referred to as Manisa/69, but this has sequence changes n151t, n152c, c204t, n237t, n238t, n239t, c825a, g840t, c882t, a912g, g932t, g1110a, c1140t, g1170a, n1287t, t1434c, t1515g, c2025t, t2061c, a2162g.
- UKG/10/2001 retains its name and sequence.
