# Supplementary material for: Tracking the Antigenic Evolution of Foot-and-Mouth Disease Virus
Source: PLoS One. 2016 Jul 22;11(7):e0159360. doi: 10.1371/journal.pone.0159360 (PMC4957747; doi:10.1371/journal.pone.0159360)
Supplement: S2 Table — The dataset shows the aligned VP2, VP3 and VP1 proteins of example SAT1 and O isolates used in the study alongside representative isolates from the other five serotypes. The four contiguous surface-exposed structural motifs confirmed as containing antigenic sites on at least four serotypes are highlighted in red–locations are approximate due to structural differences between the serotypes. The RGD cell surface receptor-binding motif, in the centre of the third site, is highlighted in blue. (DOCX) [file pone.0159360.s005.docx]

| VP2 (86-306) | | 1 2 3 4 5 |
| --- | --- | --- |
|  |  | 12345678901234567890123456789012345678901234567890 |
| 0 | SAT1 KNP/196/91 | DKKTEETTLLEDRILTTSHGTTTSTTQSSVGITYGYADSDRFLPGPNTNG |
|  | O UKG/34/2001 | DKKTEETTLLEDRILTTRNGHTTSTTQSSVGVTYGYATAEDFVSGPNTSG |
|  | A IRQ/24/64 | DKKTEETTLLEDRILTTRNGHTTSTTQSSVGVTYGYSTQEDHVSGPNTSG |
|  | SAT2 KNP/19/89 | DKKTEETTLLEDRIVTTRHGTTTSTTQSSVGVTYGYADADSFRPGPNTSG |
|  | SAT3 ZIM/5/91 | DKKTEETTLLEDRILTTRHNTTTSTTQSSVGVTYGYASADRFLPGPNTSG |
|  | Asia1 PAK/1/54 | DKKTEETTLLEDRILTTRNGHTTSTTQSSVGVTYGYAVTEDAVSGPNTSG |
|  | C SPA/S8c1/70 | DKKTEETTLLEDRILTTRNGHTTSTTQSSVGVTFGYATAEDSTSGPNTSG |
| 50 | SAT1 KNP/196/91 | LETRVEQAERFFKHKLFDWTLEQRFGTTHVLELPTDHKGIYGQLVDSHSY |
|  | O UKG/34/2001 | LETRVVQAERFFKTHLFDWVTSDPFGRCYLLELPTDHKGVYGSLTDSYAY |
|  | A IRQ/24/64 | LETRVVQAERFFKKYLFDWTPDKAFGHLEKLELPTDHKGVYGHLVDSFAY |
|  | SAT2 KNP/19/89 | LETRVQQAERFFKEKLFDWTPEKPFGTLYVLELPKDHKGIYGSLTEAYTY |
|  | SAT3 ZIM/5/91 | LETRVEQAERFFKERLFTWTASQQYAHVHLLELPTDHKGIYGAMVDNHAY |
|  | Asia1 PAK/1/54 | LETRVTQAERFFKKHLFDWTPNLAFGHCHYLELPTEHKGVYGSLMDSYAY |
|  | C SPA/S8c1/70 | LETRVHQAERFFKMALFDWVPSQNFGHMHKVVLPHEPKGVYGGLVKSYAY |
| 100 | SAT1 KNP/196/91 | IRNGWDVEVSATATQFNGGCLLVAMVPELCKLSEREKYQLTLFPHQFLDP |
|  | O UKG/34/2001 | MRNGWDVEVTAVGNQFNGGCLLVAMVPELCSIDKRELYQLTLFPHQFINP |
|  | A IRQ/24/64 | MRNGWDVEVSAVGNQFNGGCLLVAMVPEWKELTPREKYQLTLFPHQFISP |
|  | SAT2 KNP/19/89 | MRNGWDVQVTATSTQFNGGSLLVAMVPELCSLRDREEFQLSLYPHQFINP |
|  | SAT3 ZIM/5/91 | IRNGWDVQVSATSTQFNGGTLLVAMVPELHALDTRSVSQLTLFPHQFINP |
|  | Asia1 PAK/1/54 | MRNGWDIEVTAVGNQFNGGCLLVALVPELKELDTRQKYQLTLFPHQFINP |
|  | C SPA/S8c1/70 | MRNGWDVEVTAVGNQFNGGCLLVALVPEMGDISDREKYQLTLYPHQFINP |
| 150 | SAT1 KNP/196/91 | RTNTT--AHIQVPYLGVDRHDQGTRHKAWTLVVMVVAPYTNDQTIGSNKA |
|  | O UKG/34/2001 | RTNMT--AHIVVPYLGVNRYDQYKKHKPWTLVVMVVSPLTTNT-VSAGQI |
|  | A IRQ/24/64 | RTNTT--AHIQVPYLGVDRHDQGKKHKAWTLVVMVVAPYTNDQTIGSSKA |
|  | SAT2 KNP/19/89 | RTNTT--AHIQVPYLGVNRHDQGKRHQAWSLVVMVLTPLTTETQMNSGTV |
|  | SAT3 ZIM/5/91 | RTNTT--AHIVVPYIGVNRHDQVKMHKAWTLVVAVLAPLTTSN-MGQDNV |
|  | Asia1 PAK/1/54 | RTNMT--AHINVPFVGVNRYDQYALHKPWTLVVMVVAPLTVKT-GGSEQI |
|  | C SPA/S8c1/70 | RTNMT--AHITVPYVGVNRYDQYKQHRPWTLVVMVVAPLTTNT-AGAQQI |
| 200 | SAT1 KNP/196/91 | EVYVNIAPTNVYVAGEKPAKQ |
|  | O UKG/34/2001 | KVYANIAPTNVHVAGEFPSKE |
|  | A IRQ/24/64 | KVYANIAPTHVHVAGELPSKE |
|  | SAT2 KNP/19/89 | EVYANIAPTNVFVAGEKPAKQ |
|  | SAT3 ZIM/5/91 | EVYANIAPTNVFVAGEKPTKQ |
|  | Asia1 PAK/1/54 | KVYMNAAPTYVHVAGELPSKE |
|  | C SPA/S8c1/70 | KVYANIAPTNVHVAGELPSKE |

| VP3 (307-530) | | 1 2 3 4 5 |
| --- | --- | --- |
|  |  | 12345678901234567890123456789012345678901234567890 |
| 0 | SAT1 KNP/196/91 | GILPVAVSVGYGGFQNTDPKTSDPVYGHVYNPARTGLPGRFTNLLDVAEA |
|  | O UKG/34/2001 | GIFPVACSDGYGGLVTTDPKTADPAYGKVFNPPRNMLPGRFTNFLDVAEA |
|  | A IRQ/24/64 | GIVPVACSDGYGGLVTTDPKTADPVYGMVYNPPRTNYPGRFTNLLDVAEA |
|  | SAT2 KNP/19/89 | GIIPVACSAGYGGFQNTDPKTADPIYGYVYNPSRNDCHGRYSSLLDVAEA |
|  | SAT3 ZIM/5/91 | GIFPVACNDGYGGFQNTDPKTSDPIYGLVANPPRTAFPGRFTNFLDVAEA |
|  | Asia1 PAK/1/54 | GIVPVACADGYGNMVTTDPKTADPVYGKVFNPPRTNLPGRFTNFLDVAEA |
|  | C SPA/S8c1/70 | GIFPVACSDGYGNMVTTDPKTADPAYGKVYNPPRTALPGRFTNYLDVAEA |
| 50 | SAT1 KNP/196/91 | CPTLLDFN-GVPYVTTQANSGSKVLTCFDLAFGHKNLKNTFMSGLAQYYT |
|  | O UKG/34/2001 | CPTFLRFEGGVPYVTTKTD-SDRVLAQFDLSLAAKHMSNTFLAGLAQYYT |
|  | A IRQ/24/64 | CPTFLCFDDGKPYVVTRTD-EQRLLAKFDLSLAAKHMSNTYLSGIAQYYA |
|  | SAT2 KNP/19/89 | CPTFLNFD-GKPYVVTKNN-GDKVMTCFDVAFTHKVHKNTFLAGLADYYT |
|  | SAT3 ZIM/5/91 | CPTFLDFN-GTPYVKTRHNSGSKILTHIDLAFGHKSFKNTYLAGLAQYYA |
|  | Asia1 PAK/1/54 | CPTFLRFG-EVPFVKTVNS-GDRLLAKFDVSLAAGHMSNTYLAGLAQYYT |
|  | C SPA/S8c1/70 | CPTFLMFE-NVPYVSTRTD-GQRLLAKFDVSLAAKHMSNTYLAGLAQYYT |
| 100 | SAT1 KNP/196/91 | QYSGTLNLHFMYTGPTNNKAKYMVAYIPPG--THPLPETPEMESHCHHAE |
|  | O UKG/34/2001 | QYSGTINLHFMFTGPTDAKARYMIAYAPPG--ME-PPKTPEAAAHCIHAE |
|  | A IRQ/24/64 | QYSGTINLHFMFTGSTDSKARYMVAYVPPG--VETPPDTPEKAAHCIHAE |
|  | SAT2 KNP/19/89 | QYQGSLNYHFMYTGPTHHKAKFMVAYIPPGIETEKLPKTPEDAAHCYHSE |
|  | SAT3 ZIM/5/91 | QYSGSLNLHFMYTGPTQSKARFMVAYVPPG--TEPVPSTPEEAAHCYHSE |
|  | Asia1 PAK/1/54 | QYSGTMNIHFMFTGPTDAKARYMVAYVPPG--MT-PPTDPERAAHCIHSE |
|  | C SPA/S8c1/70 | QYTGTINLHFMFTGPTDAKARYMVAYVPPG--MD-APDNPEEAAHCIHAE |
| 150 | SAT1 KNP/196/91 | WDTGLNSTFTFTVPYVSAADFAYTYSDEPEQASVQGWVGVYQVTDTHEKD |
|  | O UKG/34/2001 | WDTGLNSKFTFSIPYLSAADYAYTASDAAETTNVQGWVCLFQITHGKADG |
|  | A IRQ/24/64 | WDTGLNSKFTFSIPYVSAADYAYTASDVAETTNVQGWVCIYQITHGKAEQ |
|  | SAT2 KNP/19/89 | WDTGLNSQFTFAVPYVSASDFSYTHTDTPAMATTNGWVAVYQVTDTHSAE |
|  | SAT3 ZIM/5/91 | WDTGLNSKFTFTVPYMSAADYAYTYCDEPEQASAQGWVTLYQITDTHDPD |
|  | Asia1 PAK/1/54 | WDTGLNSKFTFSIPYLSAADYAYTASDVAEATSVQGWVCIYQITHGKAEG |
|  | C SPA/S8c1/70 | WDTGLNSKFTFSIPYISAADYAYTASHEAETTCVQGWVCVYQITHGKADA |
| 200 | SAT1 KNP/196/91 | GAVVVSVSAGPDFEFRMPISPSRQ |
|  | O UKG/34/2001 | DALVVLASAGKDFELRLPVDARTQ |
|  | A IRQ/24/64 | DTLVVSVSAGKDFELRLPIDPRSQ |
|  | SAT2 KNP/19/89 | AAVVVSVSAGPDLEFRFPIDPVRQ |
|  | SAT3 ZIM/5/91 | SAVLISVSAGADFELRLPINPVTQ |
|  | Asia1 PAK/1/54 | DALVVSASAGKDFEFRLPVDARQQ |
|  | C SPA/S8c1/70 | DALVVSASAGKDFELRLPVDARQQ |

| VP1 (531-754) | | 1 2 3 4 5 |
| --- | --- | --- |
|  |  | 12345678901234567890123456789012345678901234567890 |
| 0 | SAT1 KNP/196/91 | TTSAGEGAEPVTTDASQHGGDRRTT-RRHHTDVSFLLDRFTLVGKTQDNK |
|  | O UKG/34/2001 | TTSAGESADPVTATVENYGGETQVQ-RRQHTDVSFILDRFVKVTP-KDQI |
|  | A IRQ/24/64 | TTSTGESADPVTTTVENYGGETQVQ-RRQHTDVTFIMDRFVKIQN-LNPI |
|  | SAT2 KNP/19/89 | TTSAGEGADVVTTDPSTHGGQVVEK-RRMHTDVAFVLDRFTHV-HTNKTT |
|  | SAT3 ZIM/5/91 | TTSAGEGADVVTTDVTTHGGTVDTP-RRQHTNVEFLLDRFTHIGSITAS- |
|  | Asia1 PAK/1/54 | TTTTGESADPVTTTVENYGGETQTA-RRLHTDVAFVLDRFVKFT--PKNT |
|  | C SPA/S8c1/70 | TTTTGESADPVTTTVENYGGETQVQ-RRHHTDVAFVLDRFVKVTV-SDNQ |
| 50 | SAT1 KNP/196/91 | LTLDLLQTKEKALVGAILRAATYYFSDLEVACVGD-NKWVGWTPNGAPE- |
|  | O UKG/34/2001 | NVLDLMQTPAHTLVGALLRTATYYFADLEVAVKHE--GNLTWVPNGAPET |
|  | A IRQ/24/64 | HVIDLMQTHQHGLVGALLRAATYYFSDLEIVVRHD--GNLTWVPNGAPEA |
|  | SAT2 KNP/19/89 | FNVDLMDTKDKTLVGALLRASTYYFCDLEIACVGD-HRRVYWQPNGAPR- |
|  | SAT3 ZIM/5/91 | KTIDLLETKEHTLVGALLRSATYYFCDLEVAVLGN-AKWVGWVPNGCPH- |
|  | Asia1 PAK/1/54 | QTLDLMQIPSHTLVGALLRSATYYFSDLEIALVHT--GPVTWVPNGAPKT |
|  | C SPA/S8c1/70 | HTLDVMQAHKDNIVGALLRAATYYFSDLEIAVTHT--GKLTWVPNGAPVS |
| 100 | SAT1 KNP/196/91 | LAEVGDNPVVFSKGRTTRFALPYTAPHRCLATAYNGDCKYKPTGTAPREN |
|  | O UKG/34/2001 | ALDNTTNPTAYHKAPLTRLALPYTAPHRVLATVYNGNCKYGESPVTN--- |
|  | A IRQ/24/64 | ALSNMGNPTAYPKAPFTRLALPYTAPHRVLATVYNGTGKY-SAGGMG--- |
|  | SAT2 KNP/19/89 | TTELGDNPMVFSNKGVTRFAVPYTAPHRLLSTVYNGECKYET-PVT---A |
|  | SAT3 ZIM/5/91 | TDRVEDNPVVHAKGNVTRFALPYTAPHGVLATTYNGTCKYSK-TQSVK-P |
|  | Asia1 PAK/1/54 | ALDNQTNPTAYHKQPITRLALPYTAPHRVLATVYNGKTTYGEEPT----- |
|  | C SPA/S8c1/70 | ALNNTTNPTAYHKGPVTRLALPYTAPHRVLATAYTGTTTY-TASA----- |
| 150 | SAT1 KNP/196/91 | IRGDLATLAARIAS-ETH-IPTTFNYGRIYTDTEVDVYVRMKRAELYCPR |
|  | O UKG/34/2001 | VRGDLQVLAQKAAR----TLPTSFNYGAIKATRVTELLYRMKRAETYCPR |
|  | A IRQ/24/64 | RRGDLEPLAARVAA----QLPTSFNFGAIQATTIHELLVRMKRAELYCPR |
|  | SAT2 KNP/19/89 | IRGDRAVLAAKYSN-IKHTLPSTFNFGHVAADNSVDVYYRMKRAELYCPR |
|  | SAT3 ZIM/5/91 | RRGDMAVLAQRVEGEQQRCKPTTFNFGRLLCDS-GDVYYRMKRAELYCPR |
|  | Asia1 PAK/1/54 | MRGDRAVLASKVNK----QLPTSFNYGAVKAENITEMLIRIKRAETYCPR |
|  | C SPA/S8c1/70 | -RGDLAHLTTTHAR----HLPTSFNFGAVKAETITELLVRMKRAELYCPR |
| 200 | SAT1 KNP/196/91 | PVLTHYDHGGRDRYRTAITKPVKQ |
|  | O UKG/34/2001 | PLLA-IHP-SEARHKQKIVAPVKQ |
|  | A IRQ/24/64 | PLLA-VEVSSQDRHKQKIIAPAKQ |
|  | SAT2 KNP/19/89 | PLLPAYDYASRDRFDAPIGV-EKQ |
|  | SAT3 ZIM/5/91 | PLMVRYTHT-TDRYKVALVSPAKQ |
|  | Asia1 PAK/1/54 | PLLA-LDT-TQDRRKQEIIAPEKQ |
|  | C SPA/S8c1/70 | PILP-IQP-TGDRHKQPLVAPAKQ |

**Table S2. Pan-serotypic reference alignment of FMDV**. The dataset shows the aligned VP2, VP3 and VP1 proteins of example SAT1 and O isolates used in the study alongside representative isolates from the other five serotypes. The four contiguous surface-exposed structural motifs confirmed as containing antigenic sites on at least four serotypes are highlighted in red – locations are approximate due to structural differences between the serotypes. The RGD cell surface receptor-binding motif, in the centre of the third site, is highlighted in blue.
