# Supplementary material for: Tracking the Antigenic Evolution of Foot-and-Mouth Disease Virus
Source: PLoS One. 2016 Jul 22;11(7):e0159360. doi: 10.1371/journal.pone.0159360 (PMC4957747; doi:10.1371/journal.pone.0159360)
Supplement: S3 Table — (PDF) [file pone.0159360.s006.pdf]

Common name	Residue on alignment	O	A	C	Asia1	SAT1	SAT2	SAT3	count	Serotypes
VP2 67	152	VP2 67	VP2 67	VP2 67	VP2 67	VP2 67	VP2 67	VP2 67	1	Asia1[1]
VP2 70	155	VP2 70	VP2 70	VP2 70	VP2 70	VP2 70	VP2 70	VP2 70	2	O[2,3]
VP2 71	156	VP2 71	VP2 71	VP2 71	VP2 71	VP2 71	VP2 71	VP2 71	1	O[3]
VP2 72	157	VP2 72	VP2 72	VP2 72	VP2 72	VP2 72	VP2 72	VP2 72	8	O[3–5] Asia1[1] SAT1[6*] C[7] A[8]
VP2 73	158	VP2 73	VP2 73	VP2 73	VP2 73	VP2 73	VP2 73	VP2 73	3	O[3–5]
VP2 74	159	VP2 74	VP2 74	VP2 74	VP2 74	VP2 74	VP2 74	VP2 74	4	C[2,7] Asia1[1] O*
VP2 75	160	VP2 75	VP2 75	VP2 75	VP2 75	VP2 75	VP2 75	VP2 75	1	O[3]
VP2 77	162	VP2 77	VP2 77	VP2 77	VP2 77	VP2 77	VP2 77	VP2 77	3	O[2,3] Asia1[1]
VP2 78	163	VP2 78	VP2 78	VP2 78	VP2 78	VP2 78	VP2 78	VP2 78	1	O[9]
VP2 79	164	VP2 79	VP2 79	VP2 79	VP2 79	VP2 79	VP2 79	VP2 79	6	A[2,8] C[2,7] Asia1[1] SAT2[6]
VP2 80	165	VP2 80	VP2 80	VP2 80	VP2 80	VP2 80	VP2 80	VP2 80	1	A[10]
VP2 82	167	VP2 82	VP2 82	VP2 82	VP2 82	VP2 82	VP2 82	VP2 82	1	A[11]
VP2 88	173	VP2 88	VP2 88	VP2 88	VP2 88	VP2 88	VP2 88	VP2 88	1	A[11]
VP2 130	215	VP2 130	VP2 130	VP2 130	VP2 130	VP2 130	VP2 130	VP2 130	1	O[12]
VP2 131	216	VP2 131	VP2 131	VP2 131	VP2 131	VP2 131	VP2 131	VP2 131	1	O[3]
VP2 133	218	VP2 133	VP2 133	VP2 133	VP2 133	VP2 133	VP2 133	VP2 133	2	O[2,5]
VP2 134	219	VP2 134	VP2 134	VP2 134	VP2 134	VP2 134	VP2 134	VP2 134	2	O[2,13]
VP2 190	275	VP2 188	VP2 188	VP2 188	VP2 188	VP2 188	VP2 188	VP2 188	1	O[9]
VP2 193	278	VP2 191	VP2 191	VP2 191	VP2 191	VP2 191	VP2 191	VP2 191	1	O*
VP2 194	279	VP2 191	VP2 191	VP2 191	VP2 191	VP2 192	VP2 192	VP2 192	1	O*

Table S3: (a) Residues identified as part of epitopes on structural protein VP2 across the six tested serotypes of FMDV, along with corresponding positions on all serotypes. * Identified as antigenically important for neutralisation in this study.

Common name	Residue on alignment	O	A	C	Asia1	SAT1	SAT2	SAT3	count	Serotypes
VP3 56	362	VP3 56	VP3 56	VP3 56	VP3 56	VP3 56	VP3 56	VP3 55	2	O[3*]
VP3 58	364	VP3 58	VP3 58	VP3 58	VP3 58	VP3 58	VP3 58	VP3 57	9	A[2,10] O[2–4,9] C[2,7] Asia1[1]
VP3 59	365	VP3 59	VP3 59	VP3 59	VP3 58	VP3 58	VP3 58	VP3 57	2	Asia1[1] A[10]
VP3 60	366	VP3 60	VP3 60	VP3 59	VP3 59	VP3 59	VP3 59	VP3 58	1	Asia1[1]
VP3 61	367	VP3 61	VP3 61	VP3 60	VP3 60	VP3 60	VP3 60	VP3 59	1	A[10]
VP3 71	377	VP3 70	VP3 70	VP3 69	VP3 69	VP3 70	VP3 69	VP3 69	1	A[10]
VP3 72	378	VP3 71	VP3 71	VP3 70	VP3 70	VP3 71	VP3 70	VP3 70	2	SAT1[6*]
VP3 77	383	VP3 76	VP3 76	VP3 75	VP3 75	VP3 76	VP3 75	VP3 75	2	SAT1[6*]
VP3 138	444	VP3 134	VP3 135	VP3 133	VP3 133	VP3 135	VP3 136	VP3 134	2	SAT1[6*]
VP3 142	448	VP3 138	VP3 139	VP3 137	VP3 137	VP3 139	VP3 140	VP3 138	1	A[10]
VP3 177	483	VP3 173	VP3 174	VP3 172	VP3 172	VP3 174	VP3 175	VP3 173	1	O[12]
VP3 178	484	VP3 174	VP3 175	VP3 173	VP3 173	VP3 175	VP3 176	VP3 174	1	A[14]
VP3 181	487	VP3 177	VP3 178	VP3 176	VP3 176	VP3 178	VP3 179	VP3 177	1	A[14]
VP3 198	504	VP3 194	VP3 195	VP3 193	VP3 193	VP3 195	VP3 196	VP3 194	1	A[10]
VP3 223	529	VP3 219	VP3 220	VP3 218	VP3 218	VP3 220	VP3 221	VP3 219	1	Asia1[1]

Table S3: (b) Residues identified as part of epitopes on structural protein VP3 across the six tested serotypes of FMDV, along with corresponding positions on all serotypes. * Identified as antigenically important for neutralisation in this study.

Common name	Residue on alignment	O	A	C	Asia1	SAT1	SAT2	SAT3	count	Serotypes
VP1 44	574	VP1 43	VP1 43	VP1 43	VP1 43	VP1 43	VP1 42	VP1 43	3	O[3,4,13]
VP1 45	575	VP1 44	VP1 44	VP1 44	VP1 44	VP1 44	VP1 43	VP1 44	2	O[3,4]
VP1 46	576	VP1 44	VP1 44	VP1 44	VP1 44	VP1 45	VP1 44	VP1 45	2	O[3,4]
VP1 47	577	VP1 45	VP1 45	VP1 45	VP1 45	VP1 46	VP1 45	VP1 46	1	O[3]
VP1 48	578	VP1 46	VP1 46	VP1 46	VP1 46	VP1 47	VP1 46	VP1 47	1	O[9]
VP1 50	580	VP1 48	VP1 48	VP1 48	VP1 48	VP1 49	VP1 48	VP1 48	1	O[13]
VP1 114	644	VP1 110	VP1 110	VP1 110	VP1 110	VP1 111	VP1 110	VP1 110	1	SAT1[6]
VP1 140	670	VP1 136	VP1 136	VP1 136	VP1 136	VP1 137	VP1 136	VP1 136	1	A[15]
VP1 141	671	VP1 137	VP1 136	VP1 136	VP1 137	VP1 138	VP1 137	VP1 137	1	A[15]
VP1 142	672	VP1 138	VP1 137	VP1 137	VP1 138	VP1 139	VP1 138	VP1 138	1	O*
VP1 143	673	VP1 139	VP1 138	VP1 138	VP1 139	VP1 140	VP1 138	VP1 138	3	C[16,17] A[15]
VP1 144	674	VP1 140	VP1 139	VP1 139	VP1 140	VP1 141	VP1 139	VP1 139	5	C[7,16] Asia1[1] SAT1[6]*
VP1 145	675	VP1 141	VP1 140	VP1 140	VP1 141	VP1 142	VP1 140	VP1 140	2	A[15] Asia1[1]
VP1 146	676	VP1 142	VP1 141	VP1 141	VP1 141	VP1 143	VP1 141	VP1 141	2	A[18] Asia1[1]
VP1 147	677	VP1 143	VP1 142	VP1 141	VP1 141	VP1 144	VP1 141	VP1 142	2	A[15] Asia1[1]
VP1 148	678	VP1 143	VP1 142	VP1 141	VP1 141	VP1 145	VP1 141	VP1 143	2	A[15] Asia1[1]
VP1 149	679	VP1 143	VP1 142	VP1 141	VP1 141	VP1 146	VP1 141	VP1 143	4	A[15] Asia1[1] SAT1[6]*
VP1 150	680	VP1 143	VP1 142	VP1 141	VP1 141	VP1 147	VP1 142	VP1 144	2	A[15] Asia1[1]
VP1 151	681	VP1 144	VP1 143	VP1 141	VP1 142	VP1 148	VP1 143	VP1 145	6	A[10,18]+ O[3,19,20] Asia1[1]
VP1 153	683	VP1 146	VP1 145	VP1 142	VP1 144	VP1 150	VP1 145	VP1 147	2	O[21] A[10]+
VP1 155	685	VP1 148	VP1 147	VP1 144	VP1 146	VP1 152	VP1 147	VP1 149	7	A[10,15,18]+ O[3,4,19,20]
VP1 156	686	VP1 149	VP1 148	VP1 145	VP1 147	VP1 153	VP1 148	VP1 150	4	A[10,15,18]+ O[4]
VP1 157	687	VP1 150	VP1 149	VP1 146	VP1 148	VP1 154	VP1 149	VP1 151	3	C[7,16] SAT2[22]
VP1 158	688	VP1 151	VP1 150	VP1 147	VP1 149	VP1 155	VP1 150	VP1 152	2	C[17] A[10]+
VP1 159	689	VP1 152	VP1 151	VP1 148	VP1 150	VP1 156	VP1 151	VP1 153	4	C[23] A[10,14,15]+
VP1 160	690	VP1 153	VP1 152	VP1 149	VP1 151	VP1 157	VP1 152	VP1 154	4	A[10,14,15]+ C[7]
VP1 161	691	VP1 154	VP1 153	VP1 150	VP1 152	VP1 158	VP1 153	VP1 155	4	C[16] O[3,19] A[10]+
VP1 162	692	VP1 155	VP1 154	VP1 151	VP1 153	VP1 159	VP1 154	VP1 156	2	SAT2[6] A[10]+
VP1 164	694	VP1 157	VP1 156	VP1 153	VP1 155	VP1 161	VP1 156	VP1 158	2	SAT2[22] SAT1*
VP1 165	695	VP1 157	VP1 156	VP1 153	VP1 155	VP1 161	VP1 156	VP1 159	2	SAT2[22] SAT1*
VP1 167	697	VP1 157	VP1 156	VP1 153	VP1 155	VP1 163	VP1 158	VP1 161	1	SAT2[22]
VP1 169	699	VP1 158	VP1 157	VP1 154	VP1 156	VP1 164	VP1 160	VP1 163	1	O*

Common name	Residue on alignment	O	A	C	Asia1	SAT1	SAT2	SAT3	count	Serotypes
VP1 182	712	VP1 171	VP1 170	VP1 167	VP1 169	VP1 177	VP1 173	VP1 176	1	A[10]
VP1 184	714	VP1 173	VP1 172	VP1 169	VP1 171	VP1 179	VP1 175	VP1 177	1	SAT1[6]
VP1 185	715	VP1 174	VP1 173	VP1 170	VP1 172	VP1 180	VP1 176	VP1 178	1	A[14]
VP1 186	716	VP1 175	VP1 174	VP1 171	VP1 173	VP1 181	VP1 177	VP1 179	1	SAT1[6]
VP1 209	739	VP1 196	VP1 196	VP1 192	VP1 194	VP1 204	VP1 200	VP1 202	1	SAT1*
VP1 210	740	VP1 197	VP1 197	VP1 193	VP1 195	VP1 205	VP1 201	VP1 202	2	C[2,7]
VP1 211	741	VP1 198	VP1 198	VP1 194	VP1 196	VP1 206	VP1 202	VP1 203	4	C[23] O[5]* A[8]
VP1 214	744	VP1 201	VP1 201	VP1 197	VP1 199	VP1 209	VP1 205	VP1 206	1	A[14]
VP1 216	746	VP1 203	VP1 203	VP1 199	VP1 201	VP1 211	VP1 206	VP1 208	1	A[24]
VP1 218	748	VP1 205	VP1 205	VP1 201	VP1 203	VP1 213	VP1 208	VP1 210	1	A[10]+
VP1 219	749	VP1 206	VP1 206	VP1 202	VP1 204	VP1 214	VP1 209	VP1 211	1	O[21]
VP1 220	750	VP1 207	VP1 207	VP1 203	VP1 205	VP1 215	VP1 210	VP1 212	2	O[21] SAT2[6]
VP1 221	751	VP1 208	VP1 208	VP1 204	VP1 206	VP1 216	VP1 211	VP1 213	1	O[19]
VP1 222	752	VP1 209	VP1 209	VP1 205	VP1 207	VP1 217	VP1 212	VP1 214	1	A[14]

Table S3: (c) Residues identified as part of epitopes on structural protein VP1 across all seven serotypes of FMDV, along with corresponding positions on other serotypes. * Identified as antigenically important for neutralisation in this study. $^+$ $A_{10}61$ has a deletion at residue 677 on this alignment. Serotype A numbering is relative to the other serotype A viruses without the deletion.

References

[1] Santina Grazioli, Francesca Fallacara, and Emiliana Brocchi. Mapping of antigenic sites of foot-and-mouth disease virus serotype Asia 1 and relationships with sites described in other serotypes. *The Journal of general virology*, 94(Pt 3):559–569, March 2013.

[2] Mauricio Mateu, J Hernandez, Miguel Martínez, D Feigelstock, Susan Lea, J Perez, Ernest Giralt, David Stuart, Eduardo Palma, and Esteban Domingo. Antigenic heterogeneity of a foot-and-mouth disease virus serotype in the field is mediated by very limited sequence variation at several antigenic sites. *Journal of Virology*, 68(3):1407–1417, March 1994.

[3] Jeremy Kitson, David McCahon, and Graham Belsham. Sequence analysis of monoclonal antibody resistant mutants of type O foot and mouth disease virus: evidence for the involvement of the three surface exposed capsid proteins in four antigenic sites. *Virology*, 179(1):26–34, November 1990.

[4] John Crowther, S Farias, W Carpenter, and Alan Samuel. Identification of a fifth neutralizable site on type O foot-and-mouth disease virus following characterization of single and quintuple monoclonal antibody escape mutants. *The Journal of general virology*, 74 (Pt 8):1547–1553, August 1993.

[5] S Aktas and A R Samuel. Identification of antigenic epitopes on the foot and mouth disease virus isolate O-1/Manisa/Turkey/69 using monoclonal antibodies. *Revue Scientifique Et Technique De L Office International Des Epizooties*, 19(3):744–753, 2000.

[6] Santina Grazioli, Marcella Moretti, Ilaria Barbieri, Marialuisa Crosatti, and Emiliana Brocchi. Use of monoclonal antibodies to identify and map new antigenic determinants involved in neutralisation on FMD viruses type SAT 1 and SAT 2. *Report of the Session of the Research Group of the Standing Technical Committee of the European Commission for the Control of Foot-and-Mouth Disease*, Appendix 43:287–297, 2006.

[7] Susan Lea, J Hernandez, Wendy Blakemore, Emiliana Brocchi, Stephen Curry, Esteban Domingo, Elizabeth Fry, Robin Abu Ghazaleh, Andrew King, John Newman, David Stuart, and Mauricio Mateu. The structure and antigenicity of a type C foot-and-mouth disease virus. *Structure*, 2(2):123–139, February 1994.

[8] J C Saiz, M J Gonzalez, M V Borca, F Sobrino, and D M Moore. Identification of neutralizing antigenic sites on VP1 and VP2 of type A5 foot-and-mouth disease virus, defined by neutralization-resistant variants. *Journal of Virology*, 65(5):2518–2524, May 1991.

[9] Paul V Barnett, Alan Samuel, L Pullen, D Ansell, R N Butcher, and R M Parkhouse. Monoclonal antibodies, against O1 serotype foot-and-mouth disease virus, from a natural bovine host, recognize similar antigenic features to those defined by the mouse. *The Journal of general virology*, 79 (Pt 7):1687–1697, July 1998.

[10] A Thomas, R Woortmeijer, W Puijk, and S Barteling. Antigenic sites on foot-and-mouth disease virus type A10. *Journal of Virology*, 62(8):2782–2789, August 1988.

[11] C Bolwell, A L Brown, Paul V Barnett, R O Campbell, B E Clarke, N R Parry, E J Ouldridge, F Brown, and D J Rowlands. Host cell selection of antigenic variants of foot-and-mouth disease virus. *The Journal of general virology*, 70 (Pt 1):45–57, January 1989.

[12] E Pfaff, H J Thiel, E Beck, K Strohmaier, and H Schaller. Analysis of neutralizing epitopes on foot-and-mouth disease virus. *Journal of Virology*, 62(6):2033–2040, June 1988.

[13] Paul V Barnett, Elizabeth Ouldridge, David Rowlands, Fred Brown, and Nigel Parry. Neutralizing epitopes of type O foot-and-mouth disease virus. I. Identification and characterization of three functionally independent, conformational sites. *The Journal of general virology*, 70 (Pt 6):1483–1491, June 1989.

[14] B Baxt, V Vakharia, D Moore, A Franke, and D Morgan. Analysis of neutralizing antigenic sites on the surface of type A12 foot-and-mouth disease virus. *Journal of Virology*, 63(5):2143–2151, May 1989.

[15] C Bolwell, B E Clarke, N R Parry, E J Ouldridge, F Brown, and D J Rowlands. Epitope mapping of foot-and-mouth disease virus with neutralizing monoclonal antibodies. *The Journal of general virology*, 70 (Pt 1):59–68, January 1989.

[16] M G Mateu, M A Martínez, L Capucci, D Andreu, E Giralt, F Sobrino, E Brocchi, and E Domingo. A single amino acid substitution affects multiple overlapping epitopes in the major antigenic site of foot-and-mouth disease virus of serotype C. *The Journal of general virology*, 71 (Pt 3):629–637, March 1990.

[17] M G Mateu, M A Martínez, E Rocha, D Andreu, J Parejo, E Giralt, F Sobrino, and E Domingo. Implications of a quasispecies genome structure: effect of frequent, naturally occurring amino acid substitutions on the antigenicity of foot-and-mouth disease virus. *Proceedings of the National Academy of Sciences of the United States of America*, 86(15):5883–5887, August 1989.

[18] M Mahapatra, C Seki, Sasmita Upadhyaya, Paul V Barnett, José La Torre, and David James Paton. Characterisation and epitope mapping of neutralising monoclonal antibodies to A24 Cruzeiro strain of FMDV. *Veterinary Microbiology*, 149(1-2):242–247, April 2011.

[19] Q Xie, David McCahon, John Crowther, Graham Belsham, and Kenneth McCullough. Neutralization of foot-and-mouth disease virus can be mediated through any of at least three separate antigenic sites. *The Journal of general virology*, 68 (Pt 6):1637–1647, June 1987.

[20] J Stave, J Card, D Morgan, and V Vakharia. Neutralization sites of type O1 foot-and-mouth disease virus defined by monoclonal antibodies and neutralization-escape virus variants. *Virology*, 162(1):21–29, 1988.

[21] Nigel Parry, Paul V Barnett, E J Ouldridge, David Rowlands, and Fred Brown. Neutralizing epitopes of type O foot-and-mouth disease virus. II. Mapping three conformational sites with synthetic peptide reagents. *The Journal of general virology*, 70 (Pt 6):1493–1503, June 1989.

[22] John Crowther, C Rowe, and R Butcher. Characterization of monoclonal antibodies against a type SAT 2 foot-and-mouth disease virus. *Epidemiology and Infection*, 111(2):391–406, October 1993.

[23] J Díez, M Dávila, C Escarmís, M G Mateu, J Dominguez, J J Pérez, E Giralt, J A Melero, and E Domingo. Unique amino acid substitutions in the capsid proteins of foot-and-mouth disease virus from a persistent infection in cell culture. *Journal of Virology*, 64(11):5519–5528, November 1990.

[24] J C Saiz, M J Gonzalez, D O Morgan, J L Card, F Sobrino, and D M Moore. Antigenic comparison of different foot-and-mouth disease virus types using monoclonal antibodies defining multiple neutralizing epitopes on FMDV A5 subtypes. *Virus Research*, 13(1):45–60, May 1989.
