# Supplementary material for: Tracking the Antigenic Evolution of Foot-and-Mouth Disease Virus
Source: PLoS One. 2016 Jul 22;11(7):e0159360. doi: 10.1371/journal.pone.0159360 (PMC4957747; doi:10.1371/journal.pone.0159360)
Supplement: S4 Table — (DOCX) [file pone.0159360.s007.docx]

| Capsid protein | β-sheet | SAT1 mar-mutants sorted by presence at different sites (**bold** for match). Structural numbering xyyy refers to VPx yyy. | Residues on common alignment identified by antigenic analysis, **bold** for match (structure name) |
| --- | --- | --- | --- |
| VP2 | B-C | **D2072N**; D1181A  **D2072N**; D1181G  **D2072V**; D1181Q  D1181A/G/N/Q; **D2072N/V; S3071L;** *C3076R***; E3135A/K** | **VP2 72** (2072) |
| VP3 | B-C  E-F | **S3071L**; E1179K  **S3071L**; D1181A  E1179A/K; **S3071L;** *C3076R***; E3135K**  *C3076R*; E1179A  *C3076R*; D1181A/N  D1181A/G/N/Q; **D2072N/V; S3071L;** *C3076R***; E3135A/K**  **E3135V**  **E3135A**; D1181N/G  **E3135K**; E1179A/K  D1181A/G/N/Q; **D2072N/V; S3071L;** *C3076R***; E3135A/K** | **VP3 72** (3071)  ^*^  **VP3 138** (3135) |
| VP1 | E-F  G-H  G-H  H-I | R1111G  R1111G; A2156T  R1111G; R1145C; **G1141C**; **E1146G**  R1111G; R1145C; **G1141C**; **E1146G**  **E1146G/A** & I1148T  T1154I, A1156V/T,  A1157P  E1179A/K; **S3071L;** *C3076R***; E3135K**  **S3071L**; E1179K  **S3071L**; D1181A  *C3076R*; D1181A/N  **D2072N**; D1181A  **D2072N**; D1181G  **D2072V**; D1181Q  D1181A/G/N/Q; **D2072N/V; S3071L;** *C3076R***; E3135A/K** | **VP1 144** (1141)  **VP1 149** (1146) |

**Table S4. SAT1 mar-mutants.** Data in column 3 is reproduced from (Grazioli et al, 2006). Column 4 and Table S3 give correspondence between names on structure and our common alignment. ^*^Substitution at VP3 77 on common alignment (*3076* on structure) was identified by antigenic analysis but was not significant after Holm-Bonferonni correction.
